# Peripheral and central vestibular neuromodulation improve postural control in adolescent idiopathic scoliosis: a randomized, sham-controlled, multi-arm intervention study

**DOI:** 10.1186/s12984-026-02056-w

**Published:** 2026-06-30

**Authors:** Huaqing Chen, Liping Zhao, Yuelong Li, Qiuhua Yu, Lingling Shen, Raymond Kai-Yu Tong, Shijue Li, Chunlin Chen, Hongmin Wu, Disheng Xie, Guifang Zhang, Ying-Shing Chan, Xiao-Qian Hu, Chuhuai Wang

**Affiliations:** 1 Department of Rehabilitation Medicine, The First Affiliated Hospital, Sun Yat -sen University, Guangzhou, 510080 China; 2 Guangdong Engineering and Technology Research Centre of Rehabilitation Medicine and Translation, Guangzhou, China; 3 Guangdong Provincial Clinical Research Center for Rehabilitation Medicine, Guangzhou, China; 4 Department of Biomedical Engineering, The Chinese University of Hong Kong, Shatin, Hong Kong SAR China; 5 Institute of Intelligent Manufacturing, Guangdong Academy of Sciences, Guangzhou, China; 6 School of Biomedical Sciences, Li Ka Shing Faculty of Medicine, The University of Hong Kong, Pokfulam, Hong Kong SAR China; 7 Neuroscience Research Centre, The University of Hong Kong, Pokfulam, Hong Kong SAR China

**Keywords:** Adolescent idiopathic scoliosis, Postural control, Galvanic vestibular stimulation, Transcranial direct current stimulation

## Abstract

**Background:**

Adolescent idiopathic scoliosis (AIS) features structural spinal deformity and impaired postural control, which may be associated with altered vestibular function and multisensory integration. Neuromodulatory approaches such as noisy galvanic vestibular stimulation (nGVS) and high-definition transcranial direct current stimulation (HD-tDCS) have shown promise in enhancing balance in healthy and older populations. However, whether peripheral and cortical vestibular neuromodulation exert distinct or combined effects on postural control in AIS remains unknown. This study aimed to compare the immediate post-stimulation effects of peripheral (nGVS), central (HD-tDCS), and combined (nGVS + HD-tDCS) vestibular modulation on postural control in AIS and explore their neuromuscular and cortical correlates.

**Methods:**

Forty adolescents with AIS were randomly assigned to sham, nGVS, HD-tDCS (anodal CP6), or nGVS+tDCS groups. Center of pressure (COP), surface electromyography (EMG) for bilateral gastrocnemius (GM), tibialis anterior (TA), and erector spinae (ES), and functional near-infrared spectroscopy (fNIRS) for bilateral inferior parietal lobule (IPL), superior parietal lobule (SPL), supplementary motor area (SMA), and primary motor cortex (M1) were simultaneously recorded during an eyes-closed foam-standing Romberg task to evaluate behavior, muscular and cortical responses before and after a single-session neuromodulation intervention.

**Results:**

Active interventions (nGVS, HD-tDCS, and combined nGVS + HD-tDCS) significantly reduced COP path length and sway area versus sham, with the combined nGVS + HD-tDCS demonstrating the greatest reduction in path length. EMG analysis showed reduced gastrocnemius exertion following nGVS (right side) and combined stimulation (both sides). fNIRS revealed no hemodynamic changes after nGVS, while HD-tDCS increased activation in the right IPL, and enhanced functional connectivity (FC) between the right IPL and right SMA. Combined nGVS + HD-tDCS further amplified right IPL and right SPL activity, and strengthened connectivity within bilateral SMA and between the right IPL and right SMA.

**Conclusions:**

nGVS, HD-tDCS, and their combination improve postural control in AIS through distinct neuromodulation mechanisms. Combined stimulation was associated with the largest improvement in COP path length and broader cortical network modulation.

*Trial registration* Registered at Chinese Clinical Trial Registry (ChiCTR2500110347) on July 25, 2025.

**Supplementary Information:**

The online version contains supplementary material available at https://doi.org/10.1186/s12984-026-02056-w.

## Introduction

Adolescent idiopathic scoliosis (AIS) is the most prevalent spinal deformity in adolescents, characterized by a lateral spinal curvature in the frontal plane (Cobb angle) exceeding 10°, typically emerging between ages 10 and 18 [1, 2]. Affecting 1–4% of adolescents globally [2], AIS not only induces structural asymmetry but also impairs functional mobility, diminishes quality of life, and disrupts postural control [3]. Patients often exhibit greater sway and impaired balance during both static and dynamic tasks [4–7], which could attribute to deficits in sensorimotor integration and aberrant neuromuscular coordination [8–11]. While current treatments (e.g., bracing, surgery) focus on correcting structural deformity, they fail to address postural instability or its underlying neural mechanisms [12, 13].

The vestibular system serves as the neural cornerstone of postural control, orchestrating balance through multilayered sensorimotor integration with vision and proprioception [14]. Peripheral sensory organs-the semicircular canals and otoliths-detect angular and linear acceleration of the head, transmitting signals via the vestibular nerve to the brainstem and cerebellum. These inputs converge with visual and proprioceptive afferents in the vestibular nuclei, thalamus, and cortex, forming adaptive feedback loops for posture and gaze stabilization. Critically, vestibulo-spinal pathways modulate anti-gravity muscle tone and vestibulo-thalamo-cortical projections enable spatial awareness and postural adaptation [15, 16]. Disruptions in this network, whether from peripheral hypofunction or central processing deficits, manifest as imbalance and increased fall risk [17–19].

Mounting evidence implicates vestibular dysfunction in AIS pathophysiology. Structural abnormalities were identified in the vestibular end organs of AIS patients, including altered semicircular geometry that compromise vestibular sensitivity and spatial encoding [20–22]. Neuroimaging studies have further demonstrated both gray matter alterations, including reduced cortical thickness in areas such as parieto-insular vestibular cortex [23], and white matter disruption - aberrant supramarginal gyrus connectivity within the vestibular processing network [24]. These central abnormalities likely compound peripheral vestibular deficits, creating a multilevel impairment of sensorimotor integration. Supporting this mechanistic link, animal models of developmental vestibular lesions recapitulate core AIS features, inducing asymmetric paraspinal muscle activity and progressive spinal deformities when sensory compensation fails [25, 26]. While these findings establish vestibular dysfunction as a plausible etiological factor for AIS, critical gaps remain as to whether targeted vestibular neuromodulation can restore postural control, highlighting the need for mechanism-driven intervention studies to advance evidence-based therapeutics for AIS populations.

Neuromodulation technologies permit targeted vestibular interventions to address postural control deficits in AIS. Noisy galvanic vestibular stimulation (nGVS) targets the peripheral nervous system via subthreshold electrical current, thereby enhancing vestibular signal transmission through stochastic resonance [27–29]. Clinical evidence demonstrates nGVS enhances postural stability in both healthy individuals and patients with various vestibular pathologies [30–33]. High-definition transcranial direct current stimulation (HD-tDCS) is a non-invasive neuromodulation approach that applies a weak, constant electrical current across the scalp to influence cortical excitability. These effects are polarity dependent, with anodal and cathodal stimulation typically associated with facilitatory and inhibitory effects, respectively [34, 35]. Complementarily, HD-tDCS targeting the right temporoparietal junction (anode CP6) has been shown to modulate vestibular-related cortical processing and induce lasting changes in cortical excitability consistent with neuroplasticity [36, 37]. The right temporoparietal junction, a hub for multisensory integration of vestibular and somatosensory information, plays a crucial role in spatial orientation and balance. Thus, nGVS and HD-tDCS act on distinct but complementary levels of the vestibular-postural system. nGVS enhances the quality of vestibular input at the peripheral level, while HD-tDCS facilitates cortical excitability and multisensory integration. Given their complementary mechanisms, combining peripheral and cortical stimulation may strengthen both vestibular signal transmission and integration, offering a rationale for dual-modality intervention in AIS.

This study systematically evaluated efficacy of peripheral, central and combined vestibular interventions in four cohorts (sham, nGVS, tDCS, and nGVS+tDCS) for postural control optimization in AIS by a comprehensive multimodal assessment. Using synchronized center-of-pressure (COP) measurements, surface electromyography (EMG), and functional near-infrared spectroscopy (fNIRS) recordings during challenging balance tasks, we quantitatively assess treatment effects across behavioral (postural sway), peripheral (muscle activation), and central (cortical activation) responses. As the first investigation combining peripheral and central vestibular neuromodulation in AIS with concurrent tri-modal physiological monitoring, our approach elucidates the neuromechanical mechanisms underlying postural improvements in AIS. The findings provide clinical evidence for developing targeted, physiology-guided rehabilitation protocols to address postural instability in AIS and other conditions.

We hypothesized that nGVS, tDCS, and their combined application would each induce post-stimulus improvements in postural stability in AIS, and that combined stimulation would yield stronger effects than either modality alone. We further hypothesized that these behavioral improvements would be accompanied by distinct neuromuscular and cortical adaptations, reflected by altered EMG-derived root mean square (RMS) as well as changes in fNIRS beta values for HbO (βHbO) and enhanced functional connectivity (FC) within vestibular-related cortical networks.

## Methods

### Participants

AIS patients presenting to rehabilitation medicine specialists were evaluated and screened by our research team. A total of 40 patients were prospectively enrolled and allocated equally to four groups (*n* = 10 per group). Because limited prior data were available regarding the immediate effects of combined peripheral nGVS and cortical HD-tDCS on postural control outcomes in AIS, this study was designed as an exploratory, sham-controlled, multi-arm neuromodulation study. The sample size was determined based on feasibility considerations, expected recruitment capacity, and sample sizes used in previous exploratory neuromodulation and fNIRS studies. A post hoc sensitivity analysis was performed using G*Power. With 40 participants, four groups, two repeated measurements, α = 0.05, and power = 0.80, the minimum detectable within-between interaction effect was f(V) = 0.551. Inclusion criteria were: age between 10 and 18 years, diagnosis of AIS, and a Cobb angle between 10° and 25°. Exclusion criteria included: any pre-existing orthopedic or systemic disease; history of central nervous system or neuromuscular disorders; congenital spinal anomalies; presence of metallic implants in the head; and personal or family history of epilepsy.

All participants and their legal guardians provided written informed consent prior to participation. The study protocol was registered on July 25, 2025 at Chinese Clinical Trial Registry (ChiCTR2500110347) and approved by the Ethics Committee of the First Affiliated Hospital of Sun Yat-sen University.

### Study design

**Fig. 1:**
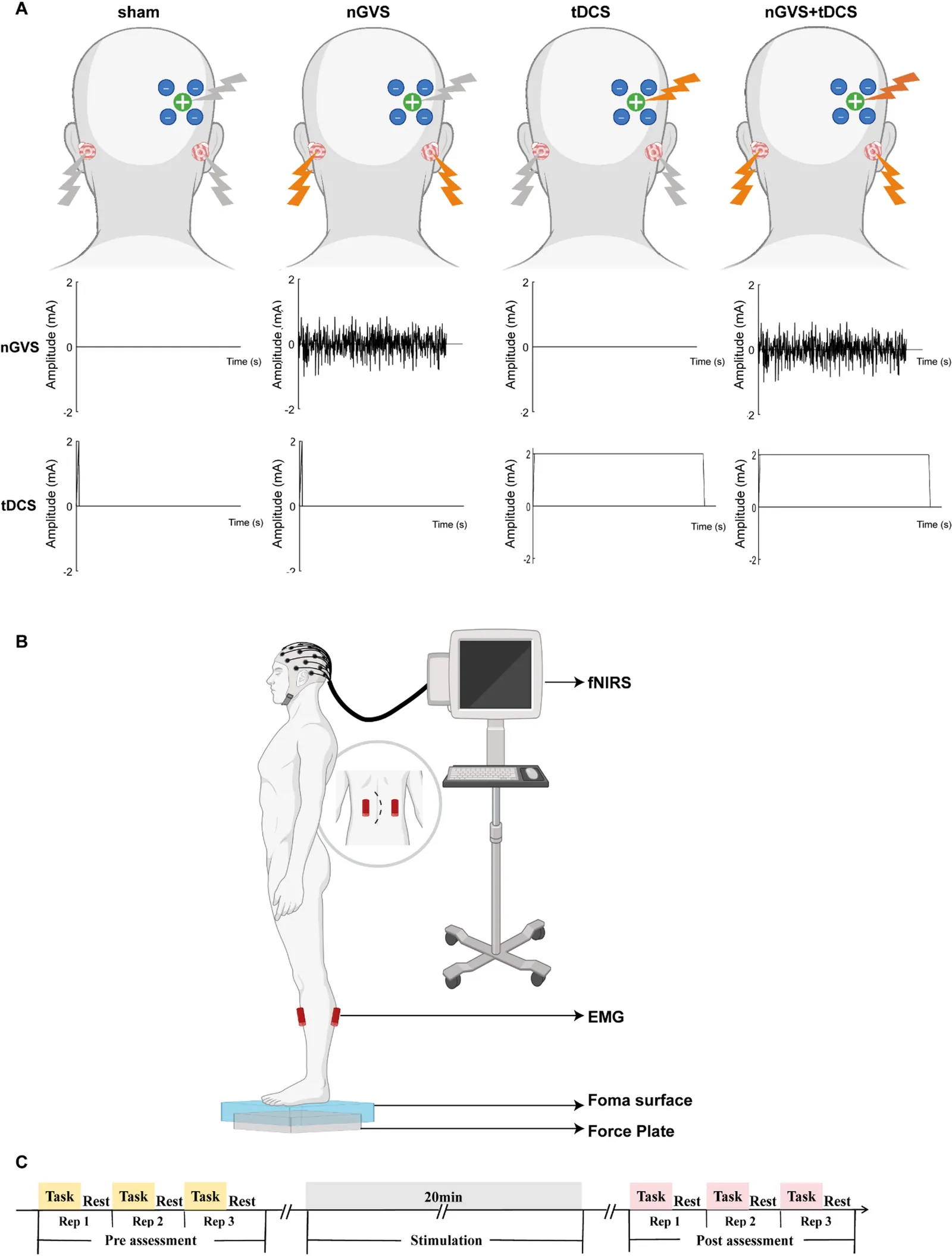
Schematic illustration of the experimental design and procedures. **A** Stimulation waveform and electrode montage for each group: sham: sham nGVS and sham tDCS; nGVS: real nGVS and sham tDCS; tDCS: sham nGVS and real tDCS; and nGVS + tDCS: real nGVS and real tDCS. **B** Task paradig: Participants performed a modified Romberg task (eyes closed standing on a foam pad placed over a force plate), while COP, EMG, and fNIRS signals were recorded. **C** Experimental timeline. Each trial consisted of a 30-s standing task followed by 30-s rest, repeated three times. Post-assessment was conducted immediately after stimulation using the same protocol as the pre-assessment

This randomized, quadruple-arm, sham-controlled trial employed triple blinding (participants, outcome assessors, data analyst) to investigate acute neuromodulatory effects on postural control in AIS. Forty eligible participants were allocated via random number table to four groups (*n* = 10/group): sham (sham nGVS and sham HD-tDCS), nGVS (real nGVS and sham HD-tDCS), tDCS (sham nGVS and real HD-tDCS), and nGVS + tDCS (real nGVS and real HD-tDCS) (Fig. 1A). To maintain blinding integrity and minimize operational bias, a clear separation of investigator roles was implemented. A designated unblinded investigator was exclusively responsible for group allocation, device configuration, and stimulation delivery. Participants remained completely unaware of their group assignments. Conversely, the outcome assessors conducting the balance, EMG, and fNIRS evaluations, as well as the data analysts performing offline processing, were entirely independent of the intervention process and strictly blinded to group allocations until all primary statistical analyses were fully completed. All outcomes were assessed during eyes-closed foam-standing Romberg task, a paradigm that challenges postural stability by removing visual input and altering somatosensory cues [38]. When visual and sensory information are restricted, balance control is largely dependent on vestibular function [39].

### Intervention protocols

The nGVS was delivered using a current stimulator (BC102-IV; BrainCLOS Co., Ltd., Shenzhen, Guangdong, China) via a pair of circular silicone gel electrodes with a surface area of 1.75 cm², placed bilaterally over the mastoid processes. For active nGVS (Groups nGVS and nGVS+tDCS), stimulation consisted of bilateral alternating current with a zero-mean Gaussian random noise waveform (bandwidth: 0.02–30 Hz; dominant spectral power: 0.02–20 Hz) at an intensity individually calibrated to 80% of the participant’s perceptual threshold. The perceptual threshold was determined using a standardized bilateral ascending staircase procedure [40]. Electrical current was initiated at a low intensity (0.1 mA) and increased in 0.05 mA increments, with each stimulation level maintained for approximately 10 s, until the participant reported a mild but reliable cutaneous sensation at the mastoid electrode sites, such as tingling or tapping. The threshold-estimation procedure was repeated three times for each participant, and the mean of the three estimates was used as the individual perceptual threshold. For active nGVS, stimulation was delivered at 80% of each participant’s perceptual threshold, without ramp-up or ramp-down phases. Because the stimulation intensity was subthreshold, active nGVS was not expected to produce perceivable cutaneous sensations. For sham nGVS (Groups sham and tDCS), electrodes were placed identically, but the stimulator delivered 0 mA throughout the entire session, also without ramp-up or ramp-down phases. Device screens and indicators were activated identically to the active condition to maintain visual blinding.

HD-tDCS was administered using a dedicated device (Neuracle, Borui Kang Medical Technologies Co., Ltd., China) with a 4 × 1 ring electrode montage cap. Each ring electrode had an outer diameter of 12 mm and an inner diameter of 6 mm, corresponding to an effective contact area of approximately 0.85 cm². The central electrode was positioned over the right parietal cortex (10–20 EEG system location CP6), surrounded by four return electrodes at C4, T8, P6, and P8. For active HD-tDCS (Groups tDCS and nGVS+tDCS), the central electrode served as the anode, delivering a constant current of 2 mA (ramped up/down over 10 s at session start/end, maintaining 2 mA for 19 min 40 s). For sham HD-tDCS (Groups sham and nGVS), stimulation ramped up to 2 mA over 10 s and immediately ramped down to 0 mA (sustained for the remaining 19 min 50 s) to successfully mimic the transient initial cutaneous sensation of active HD-tDCS without inducing sustained cortical modulation.

Participants were comfortably seated with their eyes open during the stimulation procedure. Electrodes for both nGVS and tDCS were applied according to the protocols above, specific to their assigned group. The concurrent 20-minute stimulation session commenced once both devices were initiated according to the group-specific parameters (active or sham for each modality). Participants were instructed to report any sensations experienced during the session.

### Experimental procedure

All participants underwent identical assessment protocols, including baseline measurements of center of pressure (COP) dynamics, surface EMG from postural muscles, and fNIRS cortical hemodynamics during a Romberg task (Fig. 1B, eyes closed on foam; 30 s task with 30 s rest, repeated three times). This was followed by a 20-minute group-specific neuromodulation session and immediate post-intervention reassessment (≤ 5 min post-stimulation) of the same COP, EMG, and fNIRS outcomes under matched task conditions (Fig. 1C). The experimental setup and posture adopted during the task are illustrated in Figure S1.

### Data acquisition and preprocessing

#### COP acquisition and preprocessing

Static postural stability was quantified using a clinically validated Nintendo Wii Balance Board (WBB; Nintendo, Japan) [41]. COP signals were acquired using BrainBLoX software with a sampling rate of 100 Hz [42]. During testing, participants stood barefoot on a foam pad atop the WBB while blindfolded. Participants were instructed to maintain their posture as steadily as possible during each 30-second trial. Each participant completed three trials, with a 30-second seated rest between trials. Prior to data collection, participants were given standardized instructions and allowed to perform familiarization trials. A trained examiner remained nearby at all times to ensure participant safety.

Raw COP signals underwent a standardized processing sequence in MATLAB R2022b (MathWorks, Natick, MA). To minimize the influence of initial posture adjustment and end anticipatory effects, only the middle 20 s of data (5–25 s) were extracted for analysis. The data were low-pass filtered at 20 Hz using a fourth-order zero-phase Butterworth filter to remove high-frequency noise while retaining physiological sway components, followed by mean-centered to correct baseline drift. Post-processed trajectories were evaluated for quantitative posturography using validated metrics: medio-lateral / anterior-posterior displacement range (Range ML/Range AP), medio-lateral / anterior-posterior root mean square amplitudes (RMS ML/RMS AP), medio-lateral / anterior-posterior velocities (Velocity ML / Velocity AP), total path length, and 95% confidence ellipse area.

#### EMG acquisition and preprocessing

EMG data were collected using the wireless surface EMG device (Delsys Wireless Biofeedback System, USA) sampled at 2000 Hz. Standardized skin preparation was performed at all electrode sites, involving shaving, gentle abrasion with fine-grit medical-grade sandpaper, and alcohol cleansing [43]. Sensors were positioned bilaterally over the paraspinal muscles, located 3 cm lateral to the spinous process of the apical vertebra (AV), targeting the concave-side erector spinae (ES_concave) and convex-side erector spinae (ES_convex). Additional surface EMG sensors were positioned bilaterally over the tibialis anterior (TA), and gastrocnemius (GM) of the lower limbs, with electrode placement strictly adhering to SENIAM guidelines (www.seniam.org). Sensor orientation followed muscle fiber direction.

Surface EMG signals were sampled at 2000 Hz. For each trial, a 20-second stable segment (5–25 s) was extracted for analysis. Preprocessing included mean centering, notch filtering (48–52 Hz) to remove power-line interference, and band-pass filtering (20–450 Hz). The linear metric, RMS, was calculated from the linear envelope obtained after full-wave rectification, 6-Hz low-pass filtering, and 100-point moving averaging. To enable inter-subject and inter-muscle comparisons, RMS values were normalized to each participant’s maximum voluntary isometric contraction (MVIC) [44]. MVIC testing was performed separately for each recorded muscle group using standardized manual resistance tasks. For the erector spinae (ES), participants were positioned prone on an examination table with the pelvis and lower limbs stabilized; downward manual resistance was applied at the upper thoracic/scapular level during maximal trunk extension. For the tibialis anterior (TA), participants were seated with the knee flexed at 90° and the ankle in a neutral position, performing maximal ankle dorsiflexion against manual resistance applied to the dorsal aspect of the foot. For the gastrocnemius medialis (GM), participants performed maximal ankle plantarflexion against resistance applied to the plantar surface of the forefoot, with the knee extended to preferentially activate the gastrocnemius. For each muscle, three 5-s MVIC trials were performed with 1-min rest intervals [45]. Standardized verbal encouragement was provided throughout. A 1-s stable window of the RMS signal during the plateau phase of each contraction was extracted, and the highest RMS value across the three trials defined the MVIC reference value for subsequent %MVIC normalization.

#### fNIRS signal acquisition and preprocessing

The fNIRS signals were acquired using the Nirsmart-6000A system (Huichuang Medical Equipment Co., Ltd., Danyang, China). The sampling rate was 11 Hz, and the wavelengths used were 730 nm and 850 nm. A total of 25 source probes and 25 detector probes were placed on the scalp according to the international 10–20 EEG system (Figure S2), yielding 65 measurement channels. The optode configuration was designed to cover cortical regions of interest (ROIs), including the right/left inferior parietal lobule (IPL_R/L, BA40; IPL_R: CH1, CH2; IPL_L: CH13, CH52), right/left superior parietal lobule (SPL_R/L, BA7; SPL_R: CH16, CH17; SPL_L: CH53, CH56), right/left primary motor cortex (M1_R/L, BA4; M1_R: CH30, CH32, CH65; M1_L: CH54, CH57, CH58), and right/left supplementary motor area (SMA_R/L, BA6; SMA_R: CH31, CH33, CH46, CH47; SMA_L: CH27, CH36, CH38, CH40).

A block design paradigm was used to elicit task-related cortical activation. Each session consisted of three cycles of a 30-second task block (eyes-closed standing on a foam surface) followed by a 30-second seated rest. The timing of each task–rest cycle was synchronized across the COP, EMG, and fNIRS systems, which were recorded simultaneously.

Raw intensity signals were preprocessed using NIRSPARK (Huichuang Medical Equipment Co., Ltd., China), a MATLAB-based optical imaging software. Channel quality was formally assessed prior to further processing using the built-in quality inspection module in NirSpark. The coefficient of variation (CV) was calculated for each channel, and channels with CV > 20% were excluded as poor-quality channels. Channels showing poor optical contact or signal saturation during real-time signal inspection were also excluded. The exact number and percentage of excluded channels were systematically summarized for each intervention group and are reported in Supplementary Table S8. Motion artifacts were first corrected using the automatic motion-correction module implemented in NirSpark, with the standard-deviation threshold set to 6 and the amplitude threshold set to 0.5 to eliminate transient spikes. Following motion correction, the signals were band-pass filtered at 0.01–0.2 Hz to attenuate slow baseline drifts and high-frequency physiological noise [46]. Finally, optical density changes were converted into hemoglobin concentration changes (ΔHbO and ΔHbR) using the modified Beer-Lambert law, with the differential pathlength factor set to 6.0 for both wavelengths. Task-related cortical activation was estimated using a first-level General Linear Model (GLM) for HbO signals within NirSpark. The design matrix explicitly included task regressors corresponding to the three 30-s eyes-closed foam-standing blocks, modeled according to the task markers and convolved with the default canonical hemodynamic response function (HRF) implemented in NirSpark. The interleaved 30-s seated rest blocks served as the implicit baseline. For each participant and channel, the GLM yielded β coefficients representing task-related HbO changes relative to baseline. Channel-level βHbO values were then averaged within each predefined ROI to obtain ROI-level βHbO responses. These ROI-level βHbO values were used as secondary cortical activation outcomes in subsequent statistical analyses.

FC analysis was conducted at the individual-participant level using HbO time series in NirSpark software. Task-period HbO signals corresponding to the three 30-second eyes-closed foam-standing blocks (interleaved with 30-second rest blocks) were extracted based on task markers. Task-period FC was selected to examine intervention-related changes during vestibular-dependent postural control rather than resting-state connectivity. The eyes-closed foam-standing task reduces visual input and reliable somatosensory feedback, increasing reliance on vestibular-related sensory integration. Channels within each ROI were first averaged to generate a single representative ROI-level HbO time series for each participant. FC was then computed at the individual-subject level by calculating pairwise Pearson correlation coefficients between ROI-level time series, yielding an 8 × 8 ROI-to-ROI FC matrix for each session. Diagonal and duplicate lower-triangular entries were excluded, leaving 28 unique connections. Pearson correlation coefficients were transformed using Fisher’s r-to-z transformation. All group-level statistical analyses used individual Fisher-z values. Group-level FC matrices for visualization were generated by averaging individual Fisher-z values. Because FC was measured during a block-designed task, values reflect task-state inter-regional coupling rather than resting-state intrinsic connectivity.

### Statistical analysis

All statistical analyses were performed using R software and GraphPad Prism 9 (GraphPad Software). To reduce selective emphasis and control false-positive findings, study outcomes were structured hierarchically. COP path length during the eyes-closed foam-standing task was defined as the primary outcome because it provides a global measure of postural sway magnitude and directly reflects intervention-related changes in postural stability. COP sway area was treated as a secondary supportive postural outcome because it reflects the spatial extent of postural sway. Directional COP metrics, normalized sEMG RMS values and ROI-level β-values were defined as secondary neuromuscular and cortical activation outcomes, respectively; and ROI-to-ROI FC indices were treated as exploratory neurophysiological outcomes.

Normality of continuous variables was assessed using the Shapiro-Wilk test; nonparametric sensitivity analyses on change scores were performed when assumptions were questionable. For each specific metric within these families, a 4 × 2 mixed-design repeated measures ANOVA (RM-ANOVA) was conducted, specifying Group (Sham, nGVS, tDCS, and nGVS+tDCS) as the between-subject factor and Time (Pre/Post-intervention) as the within-subject factor to examine their main effects and the Group **×** Time interaction. Effect sizes for ANOVA effects were reported as partial eta squared (η²p). To control for multiple testing within each outcome family, the *p* values for the Group × Time interaction were adjusted using the False Discovery Rate (FDR) method. For FC analyses, this included all 28 ROI-to-ROI connections. When a significant Group **×** Time interaction (FDR-corrected *p* < 0.05) was identified, post-hoc simple-effects comparisons using the pooled error term (*df* = 36) were performed to evaluate within-group Pre–Post changes, with Bonferroni correction applied for multiple comparisons. Concurrently, between-group differences in absolute clinical improvements were assessed on delta values (Δ = Post - Pre) using a one-way ANOVA followed by Tukey’s HSD post-hoc tests. Metrics without significant interaction effects after FDR correction were reported from the overarching mixed-design ANOVA only, without further post-hoc or Δ-value analyses.

To examine whether the combined intervention produced a statistical interaction between peripheral and cortical stimulation, an additional change-score 2 × 2 factorial ANOVA was performed for selected outcomes, with nGVS (No/Yes), HD-tDCS (No/Yes), and the nGVS × HD-tDCS interaction as between-subject factors. Interaction estimates and 95% confidence intervals were reported.

## Results

### Baseline characteristics and tolerability

A total of 40 adolescents with AIS were included and allocated to one of four groups. No significant between-group differences were found in age, sex, body weight, BMI, or Cobb angle (all *p* > 0.05), indicating comparable baseline characteristics across groups (Table 1). No serious adverse events occurred during or after stimulation, and no participant withdrew because of stimulation-related discomfort. A few participants reported transient tingling during real HD-tDCS stimulation, which resolved spontaneously without additional treatment. No adverse sensations were reported during real nGVS stimulation. A structured side-effect questionnaire was not administered; therefore, tolerability was summarized based on spontaneous participant reports and investigator observation.

**Table 1:** Participant demographics and baseline measures

Characteristic	Sham	nGVS	tDCS	nGVS+tDCS	*P*
**Sex**					
Male	2/20%	3/30%	3/30%	2/20%	0.91
Female	8/80%	7/70%	7/70%	8/80%	
Age	13.00 ± 2.16	13.4 ± 2.633	12.6 ± 2.716	13.7 ± 2.452	0.78
Height (cm)	160.00 ± 11.68	156.40 ± 9.62	155.40 ± 9.36	160.10 ± 11.64	0.67
Weight (kg)	42.50 ± 8.61	41.75 ± 8.22	42.05 ± 8.62	44.75 ± 10.20	0.87
BMI (kg/m²)	16.47 ± 1.97	16.93 ± 2.28	17.33 ± 2.53	17.28 ± 2.32	0.82
Cobb (°)	17.10 ± 6.03	16.4 ± 5.25	16.6 ± 4.72	17.3 ± 5.60	0.98

nGVS, noisy galvanic vestibular stimulation. tDCS, transcranial direct current stimulation. BMI, Body mass index.

### Both central and peripheral vestibular stimulation improve COP control in AIS

**Fig. 2:**
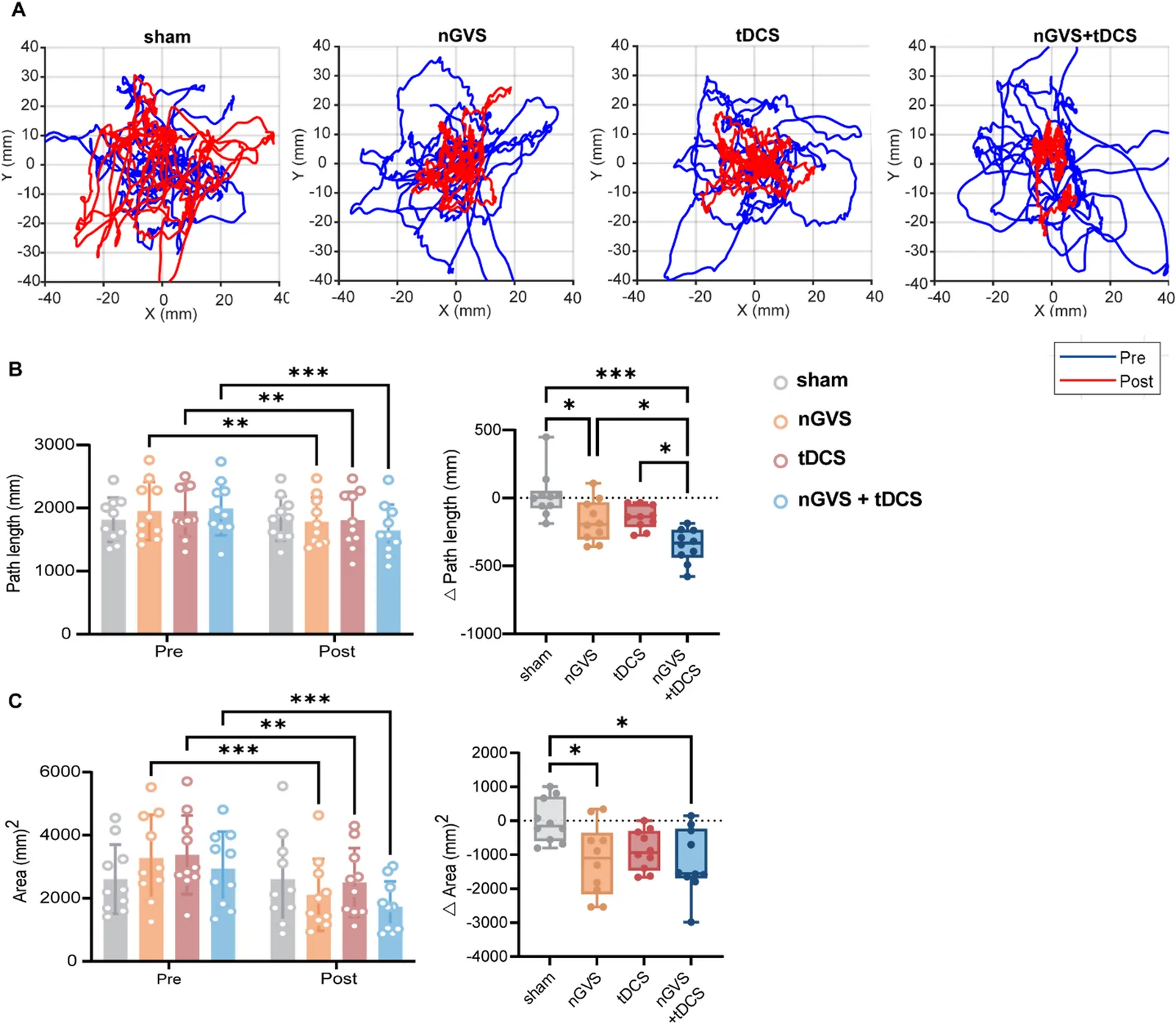
Intervention effects on COP index during the Romberg task (with eyes closed) on foam. **A** Representative COP trajectories before and after intervention in each group. Blue tracings, pre-stimulation COP trajectories. Red tracing, post-stimulation COP trajectories. **B-C** Average COP index (left) and post-hoc pairwise comparisons (right) of pre-post intervention: **B** path length and, **C** sway area. Two-way ANOVA (Group × Time) and Bonferroni-corrected post hoc tests were used for comparing pre and post COP index across groups, with data presented as mean ± SD (left). One-way ANOVA was used for pairwise comparisons of COP index changes across groups, with central line indicating the median, boxes represent the interquartile range, and lower and upper whiskers represent minimum and maximum values (right). Significant effects are indicated as * p < 0.05, ** p < 0.01, *** p < 0.001

To assess the effects of vestibular stimulation on postural stability, COP indices during the Romberg task with eye closed on foam were assessed across groups before and after intervention. Representative trajectories showed that participants receiving nGVS, tDCS, or nGVS+tDCS stimulation exhibited reduced sway after intervention, whereas minimal change was observed in the sham control (Fig. 2A).

The 4 × 2 mixed-design RM-ANOVA revealed significant interaction effects (Supplementary Table S1) for path length (*F*_(3,36)_ = 12.250, *p*_FDR < 0.001, η²*p* = 0.505), and sway area (*F*_(3,36)_ = 4.500, *p*_FDR = 0.018, η²*p* = 0.273). Regarding the secondary outcomes within the same COP family, significant interactions were also detected for Range ML (*F*_(3,36)_ = 4.507, *p*_FDR = 0.018, η²*p* = 0.273), Range AP (*F*_(3,36)_ = 8.778, *p*_FDR = 0.001, η²*p* = 0.422). In contrast, no significant interactions after FDR correction were detected for RMS ML, RMS AP, Velocity ML, or Velocity AP. Bonferroni-corrected post-hoc simple-effects tests (Table 2, Supplementary Table S2) showed that nGVS, tDCS, and nGVS+tDCS each significantly reduced path length and sway area relative to baseline, while no changes were observed in sham control (Fig. 2B-C left). Additional reductions in Range ML and Range AP were also detected in nGVS, tDCS, and nGVS+tDCS groups (Figure S3).

**Table 2:** COP, EMG, and fNIRS measurements before and after intervention in each group with post-hoc pairwise comparisons

	Variables	Group	Pre	Post	t	df	*P* (Bonferroni)
COP	Path length (cm)	sham	1814.99 ± 352.40	1836.32 ± 349.54	0.492	36	> 0.99
		nGVS	1953.00 ± 461.71	1783.61 ± 386.16	3.907	36	0.002**
		tDCS	1949.12 ± 406.39	1805.83 ± 450.43	3.305	36	0.009**
		nGVS+tDCS	1994.03 ± 428.11	1644.61 ± 409.56	8.058	36	< 0.001***
	Area (cm²)	sham	2607.53 ± 1095.74	2605.05 ± 1444.19	0.009385	36	0.99
		nGVS	3276.87 ± 1368.38	2111.70 ± 1138.10	4.419	36	< 0.001***
		tDCS	3376.31 ± 1248.38	2504.39 ± 1089.98	3.307	36	0.009**
		nGVS+tDCS	2942.10 ± 1171.16	1736.35 ± 798.45	4.573	36	< 0.001***
RMS of EMG	GM_L	sham	0.135 ± 0.064	0.156 ± 0.068	2.081	36	0.18
		nGVS	0.149 ± 0.056	0.126 ± 0.050	2.28	36	0.11
		tDCS	0.165 ± 0.083	0.150 ± 0.070	1.513	36	0.56
		nGVS+tDCS	0.150 ± 0.058	0.118 ± 0.055	3.141	36	0.01*
	GM_R	sham	0.132 ± 0.051	0.137 ± 0.047	0.6435	36	> 0.99
		nGVS	0.147 ± 0.058	0.119 ± 0.052	3.441	36	0.006**
		tDCS	0.148 ± 0.058	0.135 ± 0.048	1.711	36	0.38
		nGVS+tDCS	0.154 ± 0.058	0.122 ± 0.048	4.248	36	< 0.001***
βHbO of fNIRS	IPL_R (mmol/L)	sham	0.012 ± 0.024	0.018 ± 0.018	1.489	36	0.58
		nGVS	0.015 ± 0.029	0.020 ± 0.022	1.391	36	0.69
		tDCS	0.012 ± 0.022	0.031 ± 0.018	4.857	36	< 0.001***
		nGVS+tDCS	0.011 ± 0.028	0.033 ± 0.018	5.85	36	< 0.001***
	SPL_R (mmol/L)	sham	0.019 ± 0.021	0.016 ± 0.017	0.921	36	0.74
		nGVS	0.021 ± 0.021	0.028 ± 0.019	2.047	36	0.19
		tDCS	0.019 ± 0.022	0.028 ± 0.022	2.256	36	0.12
		nGVS+tDCS	0.019 ± 0.029	0.033 ± 0.023	4.027	36	0.001*
FC of fNIRS	IPL-R and SMA-R	sham	0.46 ± 0.17	0.43 ± 0.17	0.7652	36	0.99
		nGVS	0.45 ± 0.21	0.40 ± 0.16	1.145	36	0.99
		tDCS	0.46 ± 0.11	0.58 ± 0.14	2.928	36	0.02*
		nGVS+tDCS	0.41 ± 0.20	0.54 ± 0.22	2.933	36	0.02*
	SMA-R and SMA-L	sham	0.447 ± 0.185	0.392 ± 0.146	1.789	36	0.33
		nGVS	0.460 ± 0.136	0.513 ± 0.155	1.719	36	0.38
		tDCS	0.459 ± 0.218	0.518 ± 0.233	1.910	36	0.36
		nGVS+tDCS	0.439 ± 0.166	0.559 ± 0.219	3.869	36	0.002**

df, degrees of freedom. COP, Center of pressure. Path length, total COP path length. Area, 95% confidence ellipse area. EMG, Electromyography. RMS, Root Mean Square. Gastro_L/R, Left/Right Gastrocnemius. fNIRS, functional Near-Infrared Spectroscopy. βHbO, β coefficient for oxygenated hemoglobin changes. FC, function connection. SPL_R: right superior parietal lobule. IPL_R, right inferior parietal lobule. SMA-L/R: left/right supplementary motor area. * *p* < 0.05, ** *p* < 0.01, *** *p* < 0.001

Further between-group comparisons of post-pre differences by one-way ANOVA revealed significant group effects for path length, sway Area, Range ML and Range AP (Table 3; Supplementary Table S3). Post-hoc Tukey tests indicated that nGVS and nGVS+tDCS interventions led to significant larger reductions in sway area (Fig. 2C right), Range ML, and Range AP compared with sham (Figure S3A–B right). Post-pre differences in COP path length showed the combined nGVS+tDCS intervention achieved the greatest reduction, significantly greater than sham, nGVS, and tDCS, with nGVS alone also outperforming the sham control (Fig. 2B right). Collectively, these results demonstrate that both central (tDCS) and peripheral (nGVS) vestibular stimulation improve COP control in AIS, with combined stimulation (nGVS+tDCS) producing the most robust and widespread effects.

**Table 3:** Group comparisons of post–pre changes in COP, EMG, and fNIRS with one-way ANOVA

	Variables	∆Value(mean ± SD)				*F*	*P*	η²	Post-hoc Comparison
		sham	nGVS	tDCS	nGVS+tDCS				
COP	Path length (cm)	21.319 ± 173.837	-169.392 ± 153.969	-143.288 ± 86.554	-349.421 ± 122.839	12.05	<0.001***	0.500	∆nGVS<∆sham (*P* = 0.02*) ∆nGVS+tDCS<∆sham (*P*<0.001***) ∆nGVS+tDCS<∆nGVS (*P* = 0.03*) ∆nGVS+tDCS<∆tDCS (*P* = 0.01*)
	Area (cm²)	-2.475 ± 636.798	-1165.170 ± 1056.968	-871.922 ± 588.789	-1205.745 ± 954.940	4.5	0.009**	0.270	∆nGVS<∆sham (*P* = 0.02*) ∆nGVS+tDCS<∆sham (*P* = 0.01*)
RMS of EMG	GM_L	0.020 ± 0.045	-0.023 ± 0.022	-0.015 ± 0.019	-0.031 ± 0.032	5.266	0.004**	0.305	∆nGVS<∆sham(*P* = 0.02*) ∆nGVS+tDCS<∆sham (*P* = 0.004**)
	GM_R	0.005 ± 0.033	-0.026 ± 0.017	-0.013 ± 0.018	-0.032 ± 0.024	4.687	0.007**	0.281	∆nGVS<∆sham (*P* = 0.03*) ∆nGVS+tDCS<∆sham (*P* = 0.007**)
βHbO of fNIRS	IPL_R (mmol/L)	0.006 ± 0.010	0.005 ± 0.013	0.018 ± 0.013	0.022 ± 0.011	5.270	0.004**	0.305	∆nGVS+tDCS>∆sham (*P* = 0.020*) ∆ nGVS+tDCS>∆nGVS (*P* = 0.016*)
	SPL_R (mmol/L)	-0.004 ± 0.006	0.008 ± 0.017	0.009 ± 0.014	0.016 ± 0.010	4.207	0.012*	0.259	∆nGVS+tDCS>∆sham (*P* = 0.007**)
FC of fNIRS	IPL_R and SMA_R	-0.032 ± 0.105	-0.048 ± 0.190	0.143 ± 0.064	0.134 ± 0.128	6.343	0.002**	0.346	∆tDCS>∆sham (*P* = 0.022*) ∆nGVS+tDCS>∆sham (*P* = 0.034*) ∆tDCS>∆nGVS (*P* = 0.011*) ∆nGVS+tDCS>∆nGVS (*P* = 0.017*)
	SMA-R and SMA_L	-0.055 ± 0.076	0.053 ± 0.095	0.059 ± 0.105	0.120 ± 0.111	5.540	0.003**	0.316	∆nGVS+tDCS>∆sham(*P* = 0.002**)

COP, Center of pressure. Path length, total COP path length. Area, 95% confidence ellipse area. EMG, Electromyography. RMS, Root Mean Square. Gastro_L/R, Left/Right Gastrocnemius. fNIRS, functional Near-Infrared Spectroscopy. βHbO, β coefficient for oxygenated hemoglobin changes. FC, function connection. SPL_R: right superior parietal lobule. IPL_R, right inferior parietal lobule. SMA-L/R: left/right supplementary motor area. * *p* < 0.05, ** *p* < 0.01, *** *p* < 0.001

### EMG results—effects of peripheral vestibular stimulation (nGVS) on muscle activation

**Fig. 3:**
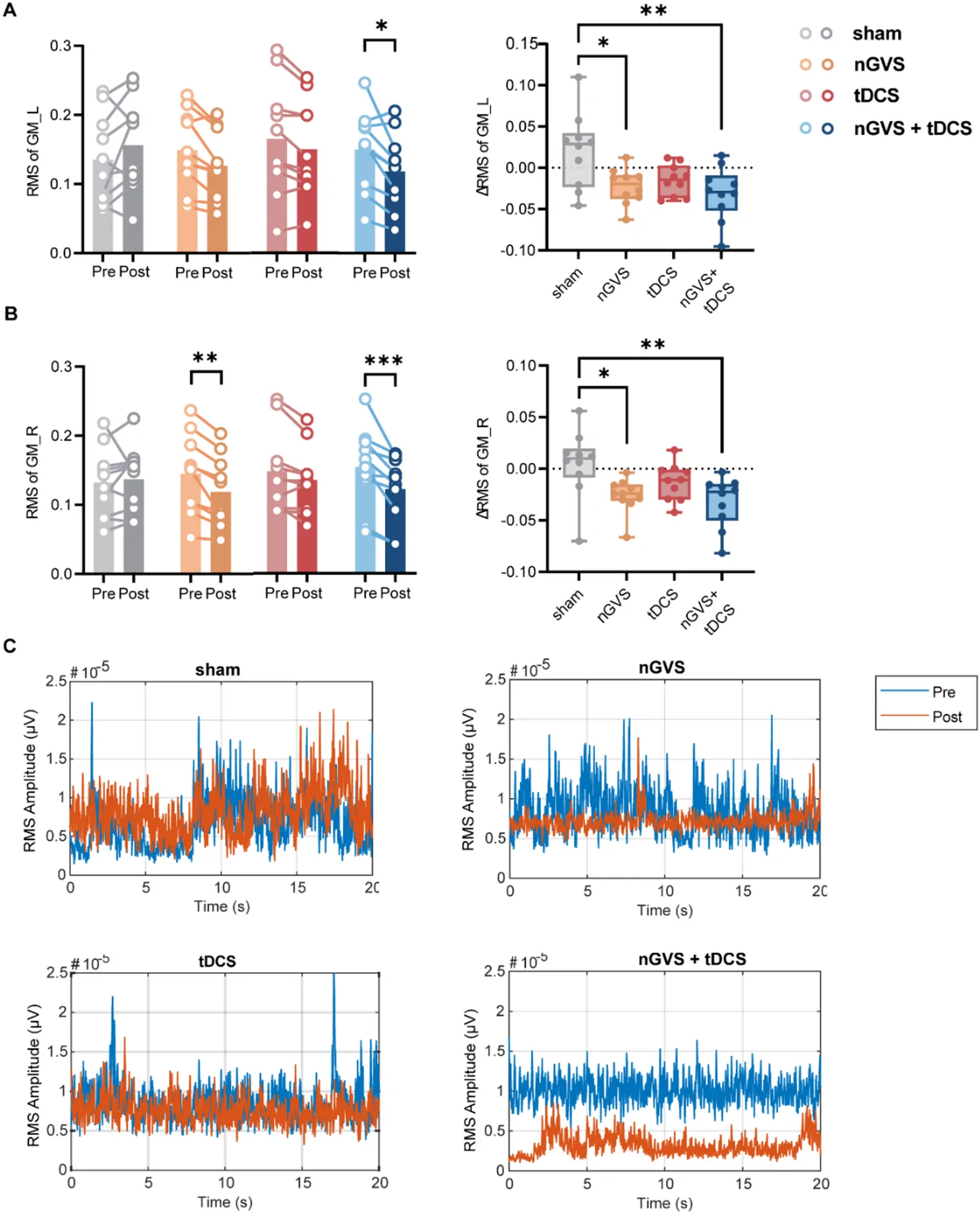
EMG amplitude in left and right gastrocnemius changes following nGVS intervention. **A-B** Root mean square (RMS) of electromyography (EMG) signals from **A** left gastrocnemius (GM_L) and **B** right gastrocnemius (GM_R) before and after intervention. Left panel, Bonferroni-adjusted simple-effects tests were used for Pre–Post within-group comparisons, with mean values shown. Right panel, between-group comparisons of RMS changes were analyzed using one-way ANOVA; box-and-whisker plots show the median (central line), interquartile range (box), and minimum–maximum values (whiskers). Significant effects are indicated as * p < 0.05, ** p < 0.01, *** p < 0.001. **C** Representative RMS waveforms of the right gastrocnemius before (in blue) and after (in red) intervention from each group

Muscle activation (RMS of EMG) during Romberg task with eyes closed on foam was evaluated before and after stimulation across groups, focusing on six key postural muscles: bilateral gastrocnemius (GM), tibialis anterior (TA), and erector spinae (ES) muscles (concave/convex). GM among postural muscles analyzed showed significant changes in activation during the tasks. Based on RMS EMG signals, two-way repeated measures ANOVA revealed significant Group × Time interactions for both the left GM (*F*_(3,36)_ = 5.266, *p*_FDR = 0.024, η²*p* = 0.305) and right GM (*F*_(3,36)_ = 4.687, *p*_FDR = 0.021, η²*p* = 0.281) after FDR correction (Supplementary Table S4), whereas no significant effects were observed in bilateral TA or ES (Supplementary Table S4; Figure S4). Model-based simple-effects comparisons following significant Group × Time interactions showed that nGVS and nGVS+tDCS significantly reduced right GM activation, while nGVS+tDCS also reduced left GM activation (Table 2; Fig. 3A-B left). In contrast, neither tDCS nor sham control produced measurable changes in GM activity. One-way ANOVA on post-pre differences (Table 3**)** confirmed significant group effects for both left GM (*F*_(3,36)_ = 5.266, *p* = 0.004) and right GM (*F*_(3,36)_ = 4.687, *p* = 0.007), with post-hoc comparisons confirming that nGVS and nGVS+tDCS produced greater reductions than the sham control for both sides (Fig. 3A-B right).

Representative RMS waveforms showed the reduced GM activity after intervention in the nGVS and nGVS+tDCS groups compared with minimal change in sham control and tDCS groups (Fig. 3C). These results demonstrate that peripheral vestibular stimulation (nGVS) plays a primary role in modulating GM activation, with the strongest effects observed when combined with central stimulation (tDCS), thereby supporting its contribution to postural control.

### fNIRS results

#### Brain activation

To simultaneously assess brain and body responses to vestibular stimulation, fNIRS was recorded during the Romberg tests before and after the intervention, together with EMG and COP measures. Cortical activation was analyzed using β values of oxygenated hemoglobin (βHbO) across eight regions of interest (ROIs), including bilateral superior parietal lobule (SPL), inferior parietal lobule (IPL), primary motor cortex (M1), and supplementary motor area (SMA). Two-way ANOVA (Group × Time) with repeated measures and FDR correction was performed, and detailed results are presented in Table 2 and Supplementary Table S5.

**Fig. 4:**
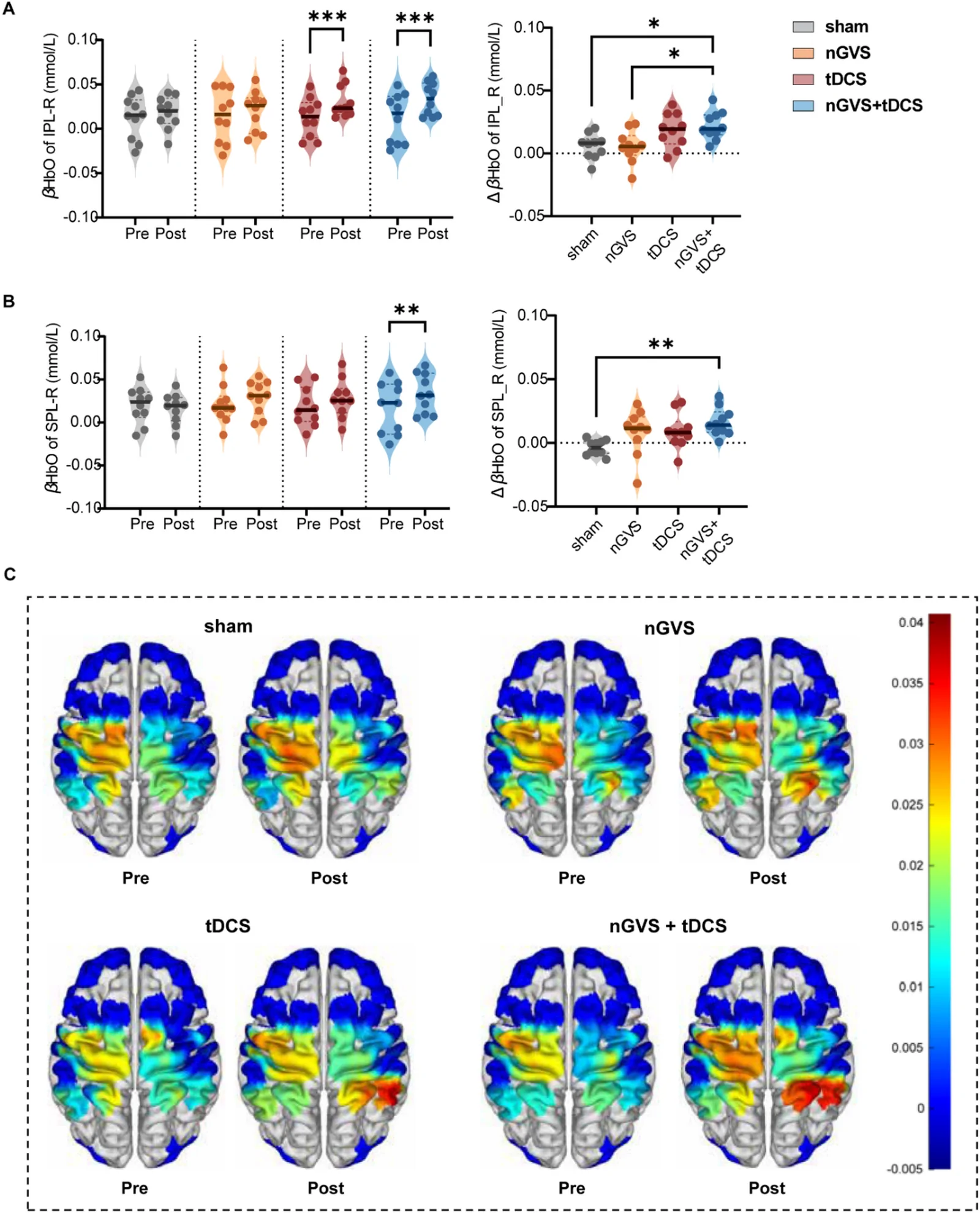
Task-related changes in cortical activation measured by fNIRS. **A, B** Changes in task-related oxygenated hemoglobin concentration (βHbO, mmol/L) in the right inferior parietal lobule (IPL-R, A) and right superior parietal lobule (SPL-R, B). Data were analyzed using two-way ANOVA (Group × Time) with Bonferroni-corrected post hoc tests and are presented as individual values with mean ± SD (left). One-way ANOVA was used for pairwise comparisons of βHbO changes across groups, with violin plots illustrating the data distribution and central line indicating the median (right). Significant effects are indicated as * p < 0.05, ** p < 0.01, *** p < 0.001. **C** Topographical brain maps illustrating cortical βHbO activation patterns before (Pre) and after (Post) intervention for each grou. Warmer colors indicate greater increases in βHbO, with the color bar representing the range of activation values

Significant corrected interaction effects (Supplementary Table S5) were observed in the right IPL (F (3,36) = 5.270, p_FDR = 0.032, η²*p* = 0.305) and right SPL (F (3,36) = 4.207, p_FDR = 0.04, η²*p* = 0.26). Post-hoc simple-effects comparisons showed that cortical activation of the right IPL increased significantly after intervention in the tDCS and nGVS+tDCS groups, whereas activation of the right SPL increased significantly in the nGVS+tDCS groups (Table 2; Fig. 4A-B left). No significant changes were observed in the other ROIs (Supplementary Table S5; Figure S5). One-way ANOVA analysis of post-pre activation changes further revealed significant group effects in both right IPL (F (3,36) = 5.27, *p* = 0.004, η²*p* = 0.305) and right SPL (F (3,36) = 4.207, *p* = 0.012, η²*p* = 0.259). For the right SPL, nGVS+tDCS produced greater activation compared with the sham control. For the right IPL, nGVS+tDCS elicited higher activation than both nGVS and sham control (Table 3; Fig. 4A-B right).

A schematic overview of cortical activation changes across groups and ROIs is shown (Fig. 4C), complementing the statistical results and suggesting that central and peripheral vestibular stimulation engage distinct, yet complementary cortical regions involved in postural control.

#### Intervention effects on functional connectivity

**Fig. 5:**
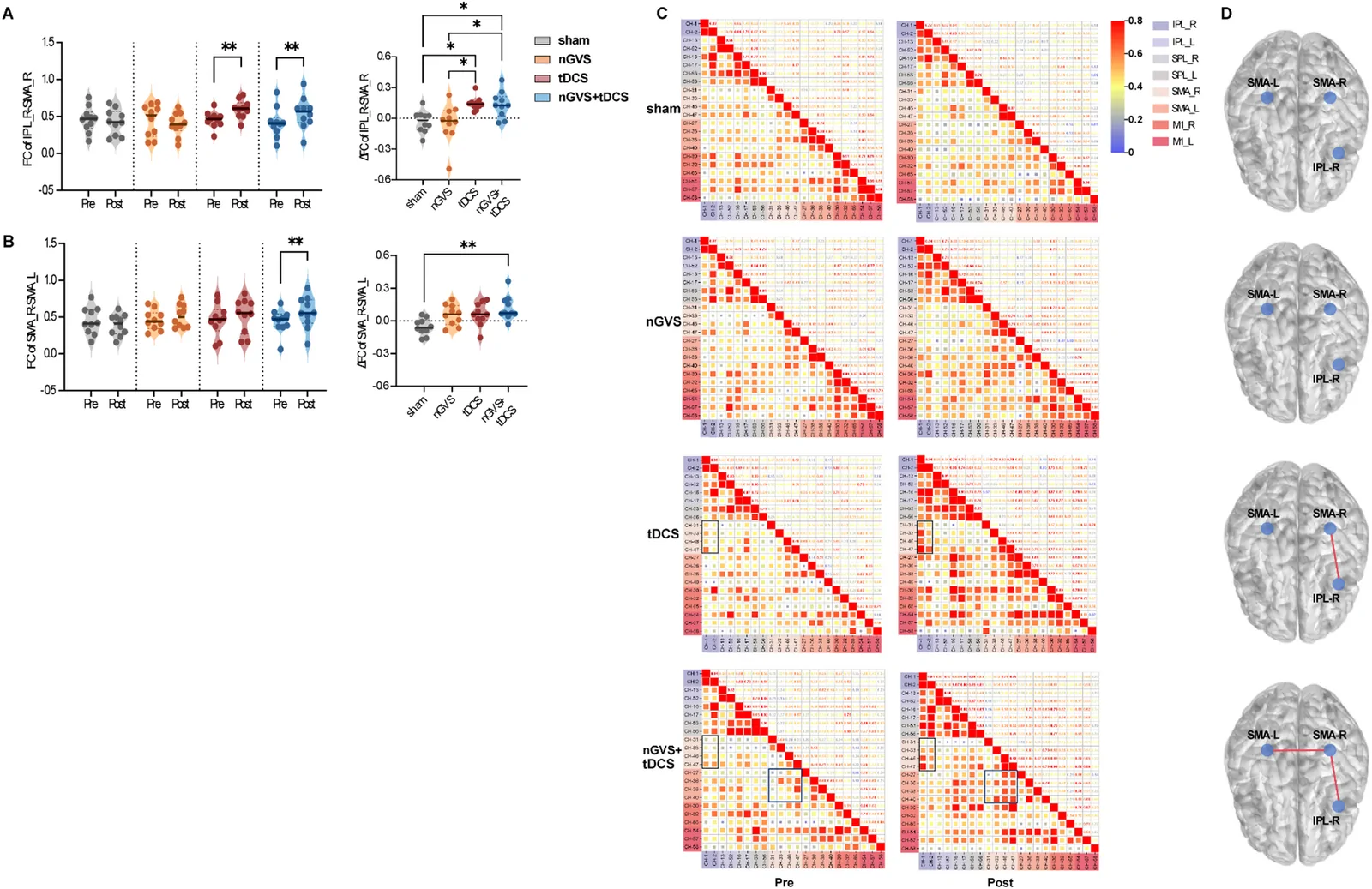
Intervention effects on functional connectivity during postural task performance. **A, B** Functional connectivity (FC) values between predefined regions of interest (ROI) before (Pre) and after (Post) intervention across groups. A, FC between the right inferior parietal lobule (IPL-R) and right supplementary motor area (SMA-R). B, FC between the right (SMA-R) and left (SMA-L) supplementary motor areas. Data are shown as individual values with mean ± SD. Two-way repeated measures ANOVA (Group × Time) with Bonferroni-corrected post hoc tests was used (left). One-way ANOVA was used for pairwise comparisons of FC changes across groups (right), with violin plots illustrating the data distribution and central line Significant effects are indicated as * p < 0.05, ** p < 0.01. **C** Group-level FC matrices illustrating connectivity patterns at pre (left panels) and post (right panels) for each intervention group. Color scale denotes correlation strength (red = stronger connectivity). **D** Brain renderings depicting ROI locations (IPL-R, SMA-R, SMA-L) and the connections showing significant post-intervention increases in FC. (p < 0.05)

To assess how vestibular stimulation modulates large-scale brain networks involved in postural control, functional connectivity (FC) was analyzed across eight cortical ROIs (bilateral SPL, IPL, M1, and SMA) before and after intervention, with 28 unique ROI pairs. By multiple comparisons with FDR corrections, two connections demonstrated significant Group × Time interactions (Supplementary Table S6): (i) IPL_R - SMA_R (*F*_(3,36)_ = 6.343, *p* _FDR = 0.028, η²*p* = 0.346) and (ii) SMA_L - SMA_R (*F*_(3,36)_ = 5.540, *p*_FDR = 0.042, η²*p* = 0.316). Post-hoc comparisons clarified that FC between the right IPL and right SMA increased significantly in the tDCS and nGVS+tDCS groups, while the right SMA and left SMA connection strengthened significantly in the nGVS+tDCS groups (Table 2; Fig. 5A-B left). None of the remaining ROI pairs showed evidence of modulation by the interventions (Supplementary Table S6). Normality of Fisher-z-transformed FC values was assessed using the Shapiro-Wilk test. All pre-intervention values were normally distributed. Only the post-intervention FC between SMA_L and M1_L showed questionable normality. For this connection, a nonparametric sensitivity analysis on the change score using the Kruskal-Wallis test was performed, which did not materially alter the main FC conclusions.

FC correlation matrices are presented, where each cell represents the group-averaged correlation value, with blue indicating weaker and red indicating stronger connectivity (Fig. 5C), with intervention-related FC increases across groups as illustrated (Fig. 5D): sham control and nGVS showed no changes, tDCS enhanced FC between the IPL_R and SMA_R, and nGVS+tDCS stimulation resulted in FC increases in both IPL_R - SMA_R and SMA_R - SMA_L.

Analyses of post - pre FC changes (Table 3) revealed significant between-group differences in the connections of IPL_R - SMA_R (*F*_(3,36)_ = 6.343, *p* = 0.002, η²*p* = 0.346) and SMA_R - SMA_L (*F*_(3,36)_ = 5.540, *p* = 0.003, η²*p* = 0.316). Both tDCS and nGVS+tDCS interventions enhanced FC in the IPL_R - SMA_R pair, when compared with the sham control and nGVS groups (Fig. 5A, right). Regarding the SMA_R - SMA_L connection, nGVS+tDCS exhibited greater increases than the sham (Fig. 5B, right). No other ROI pairs showed significant between-group differences. Together, these findings indicate that central and peripheral vestibular stimulation selectively enhance connectivity within motor–parietal and interhemispheric motor networks critical for postural control.

### Additional 2 × 2 factorial analysis of change scores

An additional change-score 2 × 2 factorial ANOVA was performed to test the nGVS × HD-tDCS interaction. For the primary outcome, COP path length, the interaction was not significant (interaction estimate = − 15.423, 95% CI: −192.816 to 161.971, *p* = 0.861; Supplementary Table S7). Similar nonsignificant interaction effects were observed for the other selected secondary and exploratory outcomes.

## Discussion

This study examined in AIS patients short-term post-stimulation effects of nGVS, right CP6-targeted HD-tDCS, and combined stimulation (nGVS + HD-tDCS) on postural control, muscle activity, and cortical activation during an eyes-closed foam-standing Romberg task. All active interventions improved postural stability relative to baseline, whereas sham stimulation induced no significant change. Combined stimulation produced the largest reduction in sway path. nGVS reduced calf muscle activation, tDCS increased cortical activation, and combined stimulation elicited both effects with additional cortical connectivity changes, indicating multi-level network reorganization. Collectively, these findings suggest that while single-modality vestibular stimulation can modulate posture-related neuromuscular and cortical processes, peripheral-central combined stimulation elicits broader and qualitatively distinct network adaptations.

Subthreshold nGVS improved postural control in AIS, consistent with previous studies in healthy and balance-impaired populations [27, 30–32, 47–49]. Our results substantiate that peripheral vestibular modulation enhances postural stabilization even in individuals with body asymmetry, such as AIS patients [50, 51]. The nGVS may improve postural control by modulating the detectability of weak vestibular afferent signals through a stochastic resonance [52, 53], thereby facilitating vestibular-related sensory integration during balance control. Because stimulation was delivered bilaterally at a subthreshold intensity, this effect is more appropriately interpreted as a non-directional enhancement of vestibular signal processing rather than as a direct correction of asymmetric vestibular inputs. However, as left-right vestibular asymmetry was not directly assessed in the present study, this mechanism remains speculative and should be examined in future studies [54]. Supported by EMG results, the observed reduction in GM RMS following nGVS indicates a more efficient postural strategy without excessive muscle recruitment. This aligns with studies in healthy subjects using similar stimulation and testing paradigms. These studies suggest that nGVS increases the excitability of the lateral vestibulospinal tract and elevates soleus H-reflex amplitudes [55, 56]. In contrast, Matsugi et al. [57] applied a suprathreshold 1 mA stimulus and observed increased soleus and TA activity, suggesting that the neuromodulatory effects of nGVS are intensity-dependent. Such discrepancies in muscle responses across studies likely arise from both population-specific and stimulation-related factors. Healthy adults with intact multisensory integration can effectively use vestibular cues and minimize muscular effort for stabilization. Conversely, AIS individuals, with reduced vestibular function and impaired sensory reweighting [58], rely more on proprioceptive feedback [3], leading to overactivation of lower-limb muscles to maintain balance [59]. In this context, nGVS may enhance vestibular signal fidelity and strengthen vestibulospinal output, thereby compensating for intrinsic sensory deficits and allowing for a more economical redistribution of muscle activity.

Previous studies have shown that nGVS can activate multisensory cortical regions, including area 2, area 3a/b, area 7, and the parieto-insular vestibular cortex (PIVC) [60, 61], as well as subcortical and vestibular-related regions such as the posterolateral thalamus, cerebellar vermis, posterior insula, and hippocampus [62, 63]. However, nGVS effects measured by fNIRS in this study revealed no significant cortical activation or functional connectivity changes across eight ROIs after FDR correction. This may be attribute to the subthreshold stimulation intensity and the lower sensitivity of fNIRS compared with fMRI for detecting subtle vestibular activations [30, 64]. Taken together, these results suggest that the nGVS effects in AIS primarily manifest through motor outputs, with limited cortical involvement likely dependent on stimulation parameters (e.g., intensity and waveform) and measurement setup.

Anodal HD-tDCS over CP6 significantly improved postural stability post stimulation, suggesting that cortical neuromodulation facilitates balance control. This aligns with previous reports showing that CP6 stimulation reduced postural instability on unstable surfaces [39] or during sensory conflicts by virtual reality immersion [65], while parietal stimulation enhanced postural adaptation by refining internal representations of body orientation and verticality [66]. AIS is associated with abnormal sensory reweighting, particularly in the integration and prioritization of vestibular, somatosensory, and visual inputs [58]. AIS is associated with impaired sensory reweighting and multisensory integration during balance control [67–70]. Notably, fNIRS revealed that HD-tDCS in AIS enhances activation in the right inferior parietal lobule (IPL), a key hub for multisensory integration [71–74]. IPL involvement likely represents a compensatory mechanism following HD-tDCS in AIS population, contributing to better sensory reweighting and neural estimation of body position in space, especially under challenging sensory conditions. Furthermore, HD-tDCS-induced enhanced functional connectivity between the right IPL and the supplementary motor area (SMA) suggests strengthened coordination between multisensory processing and motor planning. Within established vestibulo-parietal-motor pathways [17], vestibular signals ascend via the thalamus to the parieto-insular vestibular cortex and IPL, where multisensory information is integrated and relayed to the SMA and premotor cortex for anticipatory and corrective postural control [69, 75]. Strengthened IPL-SMA coupling may thus facilitate the translation of integrated sensory inputs into motor commands, providing a neural substrate for the observed behavioral improvements. Together, these findings suggest that HD-tDCS over CP6 reinforces vestibulo-parietal-motor networks, partially compensating for AIS-related deficits in multisensory integration and thereby improving postural stability.

The combined intervention produced the largest improvement in the primary postural outcome (COP path length) and uniquely recruited the right SPL together with strengthened bilateral SMA connectivity, indicating system-level optimization of the postural control network. nGVS may contribute to improved postural stability through enhancement of vestibular signal processing and vestibulospinal function [76, 77], whereas parietal HD-tDCS modulates cortical excitability and promotes integration within distributed cortical networks [78]. When combined, bottom-up vestibular afferents and top-down parietal modulation may converge to induce a metaplastic state, referring to a condition in which prior neural activity changes the brain’s readiness for subsequent plastic adaptation, thereby increasing neural responsiveness and facilitating adaptive reorganization [79]. The SPL is actively involved in vestibular-dependent spatial representation and body-centered reference frames [80, 81], while the SMA mediates anticipatory postural adjustments and bilateral motor coordination [82, 83]. This network interaction may underlie the remarkable improvements in postural control following combined vestibular stimulation, holding translational relevance not only for AIS but also for broader populations with postural control deficits.

Although the present study focused on the eyes-closed foam-standing condition to emphasize vestibular-dependent postural control, balance regulation in daily life relies on the integration and reweighting of vestibular, visual, and proprioceptive inputs. Therefore, the clinical relevance of GVS-based neuromodulation may extend beyond enhancing vestibular signals alone. Prior evidence suggests that GVS can improve balance under both eyes-open and eyes-closed conditions and may influence the relative weighting of visual cues during balance-related sensorimotor processing [84]. Complementarily, high-intensity GVS, when used to interfere with vestibular signal transmission during the observation of balance-related actions, has been shown to alter sensorimotor representations of observed actions and increase the relative weighting of visual cues [85]. These findings support the possibility that combining peripheral vestibular stimulation with cortical modulation of multisensory integration areas may facilitate adaptive sensory reweighting within the broader postural control network. Future studies should test this hypothesis under different visual and somatosensory conditions.

## Limitations

Several limitations should be acknowledged. First, the relatively small sample size of 10 participants per group may limit statistical power for between-group comparisons, particularly given the multimodal outcomes assessed. As this study was designed as an exploratory, multi-arm neuromodulation trial, the sample size was primarily determined by logistical feasibility. The post hoc sensitivity analysis indicated that the present sample was mainly able to detect relatively large within-between interaction effects; therefore, subtle or smaller neuromodulatory effects may have been missed. Larger confirmatory trials with formal a priori sample-size calculations based on predefined primary outcomes are warranted to validate these preliminary findings. Second, the effects of nGVS may be population dependent. Prior studies reported no clear stochastic resonance effect of noisy GVS on standing balance in young healthy adults [86], whereas sustained balance improvements were observed in older adults [77]. These differences suggest that responsiveness to nGVS may depend on baseline sensory function, age, and clinical status. Therefore, the present findings in AIS require cautious interpretation when extending them to other populations with balance disorders. Third, FC was estimated during a block-designed postural task, and the observed correlations may partly reflect shared task-evoked hemodynamic responses rather than purely intrinsic inter-regional connectivity. Although the same task was used before and after intervention, future studies combining task-state and resting-state FC or using GLM-residual-based connectivity are needed to better distinguish task-evoked coactivation from inter-regional coupling. Finally, tolerability and blinding integrity were not quantified using structured post-intervention questionnaires. Although no serious adverse events or stimulation-related dropouts were observed, future trials should incorporate standardized side-effect checklists and forced-choice blinding assessment questionnaires to better evaluate safety, expectancy, and placebo effects.

## Conclusion

Together, these findings reveal that peripheral and cortical vestibular neuromodulation act through complementary mechanisms to enhance postural control. nGVS primarily amplifies vestibulospinal signaling to fine-tune motor outputs, while parietal HD-tDCS optimizes cortical integration and sensory reweighting. Concurrent modulation unites the bottom-up and top-down pathways, inducing large-scale network plasticity that supports efficient sensorimotor coordination. These findings provide a mechanistic framework for vestibular-based neurorehabilitation. Beyond AIS, such combined interventions may hold translational value for other neurological or aging-related conditions characterized by deficits in multisensory integration and postural stability. Future studies with larger cohorts, diverse patient populations, and longitudinal follow-up are warranted to validate these effects and determine whether the observed benefits persist beyond the immediate post-stimulation period.

## Supplementary Information

Below is the link to the electronic supplementary material.

Supplementary Material 1.

## Data Availability

De-identified individual participant data will be made available after publication upon reasonable request for non-commercial academic research with a clearly defined research question. Access to the data requires approval from the requesting researcher’s institutional ethics committee. The de-identified datasets will be provided via a secure data transfer system.

## Abbreviations

AIS: Adolescent idiopathic scoliosis
nGVS: Noisy galvanic vestibular stimulation
tDCS: Transcranial direct current stimulation
HD-tDCS: High-definition transcranial direct current stimulation
COP: Center of pressure
EMG: Electromyography
fNIRS: Functional near-infrared spectroscopy
IPL: Inferior parietal lobule
RMS: Root mean square
SPL: Superior parietal lobule
SMA: Supplementary motor area
M1: Primary motor cortex
FC: Functional connectivity
Range AP: Anterior-posterior displacement range
Range ML: Medio-lateral displacement range
RMS AP: Anterior-posterior root mean square
RMS ML: Medio-lateral root mean square
Velocity AP: Anterior-posterior velocities
Velocity ML: Medio-lateral velocities
AV: Apical vertebra
ES_concave: Concave-side erector spinae
ES_convex: Convex-side erector spinae
TA: Tibialis anterior
GM: Gastrocnemius
MVIC: Maximum voluntary isometric contraction
ROIs: Regions of interest
