# Supplementary material for: Peripheral and central vestibular neuromodulation improve postural control in adolescent idiopathic scoliosis: a randomized, sham-controlled, multi-arm intervention study
Source: J Neuroeng Rehabil. 2026 Jun 30;23:272. doi: 10.1186/s12984-026-02056-w (PMC13584406; doi:10.1186/s12984-026-02056-w)
Supplement: Supplementary file 1 [file 12984_2026_2056_MOESM1_ESM.docx]

**Supplementary Information**

**Fig.S1.** Data acquisition during the postural assessment task.

Representative photographs show the eyes-closed standing-on-foam task from the (A) anterior, (B) posterior, and (C) lateral views, with EMG electrodes on the target muscles and fNIRS optodes on the scalp.

**Fig. S2. fNIRS measurement images.**

The positions of light sources (S1–S24, S28) and detectors (D1–D24, D28) are shown, producing 65 fNIRS channels (CH1–CH65) covering bilateral cortical regions. Dashed rectangles indicate the eight regions of interest (ROIs) selected for analysis. The triangle at the bottom represents the nasion (nose) direction. IPL_R, right inferior parietal lobule; IPL_L, left inferior parietal lobule; SPL_R, right superior parietal lobule; SPL_L, left superior parietal lobule; M1_R, right primary motor cortex; M1_L, left primary motor cortex; SMA_R, right supplementary motor area; SMA_L, left supplementary motor area

**Fig. S3.** **Intervention effects on COP index during the Romberg task with eyes closed on foam.**

(A-F) Group comparisons before and after intervention are shown (left), while pre–post differences (Δ values) for outcomes with interaction effects are illustrated (right). For outcomes without interactions, only the left panel is displayed. **(A)** medio-lateral range (Range ML), **(B)** anterior-posterior range (Range AP), **(C)** medio-lateral root mean square (RMS ML), **(D)** anterior-posterior root mean square (RMS AP), **(E)** medio-lateral velocity (Velocity ML), (**F**) anterior-posterior velocity (Velocity AP) . RM-ANOVA (Group × Time) and Bonferroni-corrected post hoc tests were used for comparing pre and post COP index across groups, with data presented as mean ± SD (left). One-way ANOVA was used for pairwise comparisons of COP index changes across groups, with central line indicating the median, boxes represent the interquartile range, and lower and upper whiskers represent minimum and maximum values (right). Significant effects are indicated as * *p* < 0.05, ** *p* < 0.01, *** *p* < 0.001.

**Fig. S4. EMG amplitude in the remaining muscles before and after interventions.**

**(A-D)** The comparison of Root mean square (RMS) of electromyography (EMG) (Mean ± SD) from **(A)** left tibialis anterior (TA_L), **(B)** right tibialis anterior (GM_R), (**C**) convex side erector spinae (convex ES), and (**D**) concave side erector spinae (concave ES) before and after intervention between groups. Significant effects are indicated as * *p* < 0.05, ** *p* < 0.01, *** *p* < 0.001.

**Fig. S5.** **Task-related cortical activation changes in the remaining** regions of interest **(ROIs) measured by fNIRS.**

**(A-F)** Between-group comparison of task-related oxygenated hemoglobin concentration (βHbO, mmol/L) following intervention in the left primary motor cortex (M1_L, A), right primary motor cortex (M1_R, B), left inferior parietal lobule (IPL_L, C), right inferior parietal lobule (IPL_R, D), left superior parietal lobule (SPL-L, E) and right supplementary motor area (SMA-R, F). Data were analyzed using RM ANOVA (Group × Time) with Bonferroni-corrected post hoc tests and are presented as individual values with mean ± SD. Significant effects are indicated as * *p* < 0.05, ** *p* < 0.01, *** *p* < 0.001.

**Supplementary Table S1**

**RM-ANOVA results for the effects of intervention on COP.**

| **COP** | **ANOVA table** | **SS** | **DFn, DFd** | **MS** | ***F*** | ***P*** | **partial *η²*** | ***P*_adj (FDR)** |
| --- | --- | --- | --- | --- | --- | --- | --- | --- |
| Path length | Group x Time | 345622 | 3, 36 | 115000.0 | 12.25 | <0.001 | 0.505 | <0.001*** |
|  | Time | 513252 | 1, 36 | 513000.0 | 54.60 | <0.001 | 0.603 |  |
|  | Group | 52049 | 3, 36 | 17400.0 | 0.05 | 0.980 | 0.004 |  |
| Area | Group x Time | 4693420 | 3, 36 | 1564473 | 4.50 | 0.009 | 0.273 | 0.018* |
|  | Time | 13165058 | 1, 36 | 13165058 | 37.87 | <0.001 | 0.513 |  |
|  | Group | 3693093 | 3, 36 | 1231031 | 0.50 | 0.680 | 0.04 |  |
| Range ML | Group x Time | 1300 | 3, 36 | 433.5 | 4.51 | 0.009 | 0.273 | 0.018* |
|  | Time | 4192 | 1, 36 | 4192 | 43.59 | <0.001 | 0.548 |  |
|  | Group | 1295 | 3, 36 | 431.5 | 1.45 | 0.240 | 0.108 |  |
| Range AP | Group x Time | 974.4 | 3, 36 | 324.8 | 8.78 | <0.001 | 0.422 | 0.001** |
|  | Time | 1256 | 1, 36 | 1256 | 33.94 | <0.001 | 0.485 |  |
|  | Group | 1048 | 3, 36 | 349.3 | 0.89 | 0.46 | 0.069 |  |
| RMS ML | Group x Time | 22.93 | 3, 36 | 7.642 | 2.88 | 0.049 | 0.121 | 0.080 |
|  | Time | 67 | 1, 36 | 67 | 25.26 | <0.001 | 0.437 |  |
|  | Group | 37.18 | 3, 36 | 12.39 | 1.27 | 0.3 | 0.048 |  |
| RMS AP | Group x Time | 13.39 | 3, 36 | 4.465 | 1.53 | 0.22 | 0.194 | 0.251 |
|  | Time | 38.69 | 1, 36 | 38.69 | 13.26 | <0.001 | 0.412 |  |
|  | Group | 35.77 | 3, 36 | 11.92 | 0.60 | 0.62 | 0.096 |  |
| Velocity ML | Group x Time | 494 | 3, 36 | 164.7 | 2.53 | 0.07 | 0.174 | 0.093 |
|  | Time | 1539 | 1, 36 | 1539 | 23.67 | <0.001 | 0.397 |  |
|  | Group | 244 | 3, 36 | 81.35 | 0.35 | 0.79 | 0.028 |  |
| Velocity AP | Group x Time | 199.8 | 3, 36 | 66.6 | 0.97 | 0.42 | 0.075 | 0.42 |
|  | Time | 735.3 | 1, 36 | 735.3 | 10.67 | 0.002 | 0.229 |  |
|  | Group | 268.7 | 3, 36 | 89.56 | 0.16 | 0.92 | 0.013 |  |

COP:Center of pressure, Range ML/AP: medio-lateral and antero-posterior range, RMS ML/AP: root mean square of COP in medio-lateral and antero-posterior direction, Velocity ML/AP: mean velocity in medio-lateral and antero-posterior direction. * *p* < 0.05, ** *p* < 0.01, *** *p* < 0.001

**Supplementary Table S2**

**Post-hoc pair-wise comparison of COP before and after intervention in each group.**

| **COP** | **Group** | **Pre** | **Post** | **t** | **df** | ***P*(Bonferroni)** |
| --- | --- | --- | --- | --- | --- | --- |
| Range ML | sham | 60.47±14.54 | 58.10±15.73 | 0.540 | 36 | 0.592 |
|  | nGVS | 72.13±16.12 | 49.42±14.06 | 5.177 | 36 | ＜0.001*** |
|  | tDCS | 75.15±9.82 | 63.11±11.71 | 2.746 | 36 | 0.009** |
|  | nGVS+tDCS | 70.12±15.62 | 49.32±13.43 | 4.741 | 36 | ＜0.001*** |
| Range AP | sham | 69.00±18.11 | 71.45±19.27 | 0.899 | 36 | 0.374 |
|  | nGVS | 73.83±12.99 | 64.71±9.90 | 3.351 | 36 | 0.002** |
|  | tDCS | 76.96±13.51 | 69.12±11.37 | 2.881 | 36 | 0.007** |
|  | nGVS+tDCS | 71.72±17.13 | 54.53±12.10 | 6.320 | 36 | ＜0.001*** |

* *p* < 0.05, ** *p* < 0.01, *** *p* < 0.001

Range ML/AP: medio-lateral and antero-posterior range, RMS ML: root mean square of COP in medio-lateral.

**Supplementary Table S3**

**One-way ANOVA of post–pre differences in COP parameters among intervention groups.**

| **COP** | **∆Value(mean±SD）** | | | | **F (DFn, DFd)** | ***P*** | ***η²*** | **Post-hoc Comparison** |
| --- | --- | --- | --- | --- | --- | --- | --- | --- |
|  | **sham** | **nGVS** | **tDCS** | **nGVS+tDCS** |  |  |  |  |
| Range ML | -2.87±7.98 | -15.88±9.10 | -12.04±9.47 | -17.27±13.36 | 4.507 | 0.008 | 0.27 | ∆nGVS＜∆sham (P=0.012*)  ∆nGVS+tDCS＜∆sham (P=0.026*) |
| Range AP | 2.45±7.74 | -9.12±7.96 | -7.837±9.73 | -17.19±8.83 | 8.778 | ＜0.001 | 0.42 | ∆nGVS＜∆sham (P=0.024*) ∆nGVS+tDCS＜∆sham (P＜0.001***) |

Δ values are expressed as mean ± SD, n.s.: not significant. * *p* < 0.05, ** *p* < 0.01, *** *p* < 0.001

**Supplementary Table S4**

**RM-ANOVA results for the effects of intervention on RMS of EMG.**

| **Muscle** | **ANOVA table** | **SS** | **DFn, DFd** | **MS均方** | ***F*** | ***P*** | **partial *η²*** | ***P*_adj (FDR)** |
| --- | --- | --- | --- | --- | --- | --- | --- | --- |
| GM_R | Group x Time | 3.99E-03 | 3, 36 | 1.33E-03 | 4.687 | 0.007 | 0.281 | 0.021* |
|  | Time | 5.44E-03 | 1, 36 | 5.44E-03 | 19.170 | <0.001 | 0.347 |  |
|  | Group | 1.07E-03 | 3, 36 | 3.56E-04 | 0.068 | 0.98 | 0.006 |  |
| GM_L | Group x Time | 7.83E-03 | 3, 36 | 2.61E-03 | 5.266 | 0.004 | 0.305 | 0.024* |
|  | Time | 2.92E-03 | 1, 36 | 2.92E-03 | 5.891 | 0.020 | 0.141 |  |
|  | Group | 6.54E-03 | 3, 36 | 2.18E-03 | 0.285 | 0.836 | 0.023 |  |
| TA_L | Group x Time | 4.08E-04 | 3, 36 | 1.36E-04 | 0.791 | 0.507 | 0.062 | 0.51 |
|  | Time | 3.70E-07 | 1, 36 | 3.70E-07 | 0.002 | 0.963 | 0.001 |  |
|  | Group | 1.39E-03 | 3, 36 | 4.65E-04 | 0.248 | 0.862 | 0.020 |  |
| TA_R | Group x Time | 6.71E-04 | 3, 36 | 2.24E-04 | 1.161 | 0.338 | 0.088 | 0.408 |
|  | Time | 9.79E-05 | 1, 36 | 9.79E-05 | 0.508 | 0.480 | 0.014 |  |
|  | Group | 1.72E-03 | 3, 36 | 5.74E-04 | 0.251 | 0.860 | 0.020 |  |
| ES_Convex | Group x Time | 6.53E-04 | 3, 36 | 2.18E-04 | 2.201 | 0.100 | 0.155 | 0.2 |
|  | Time | 2.77E-04 | 1, 36 | 2.77E-04 | 2.807 | 0.100 | 0.072 |  |
|  | Group | 2.89E-03 | 3, 36 | 9.65E-04 | 0.744 | 0.530 | 0.058 |  |
| ES_Concave | Group x Time | 2.18E-04 | 3, 36 | 7.27E-05 | 1.836 | 0.16 | 0.133 | 0.24 |
|  | Time | 6.27E-04 | 1, 36 | 6.27E-04 | 15.83 | <0.001 | 0.305 |  |
|  | Group | 4.17E-04 | 3, 36 | 1.39E-04 | 0.155 | 0.93 | 0.013 |  |

ES_Convex/Concave: Erector spinae muscle on the convex/concave side of the apical vertebra, TA_L/R: left/right tibialis anterior. * *p* < 0.05, ** *p* < 0.01, *** *p* < 0.001

**SupplementaryTable S5**

**RM-ANOVA results for the effects of intervention on βHbO of fNIRS.**

| **ROI** | **ANOVA table** | **SS** | **DFn, DFd** | **MS** | ***F*** | ***P*** | **partial *η²*** | ***P*_adj (FDR)** |
| --- | --- | --- | --- | --- | --- | --- | --- | --- |
| IPL-R | Group x Time | 1.12E-03 | 3, 36 | 3.74E-04 | 5.270 | 0.004 | 0.305 | 0.032* |
|  | Time | 3.27E-03 | 1, 36 | 3.27E-03 | 46.150 | <0.001 | 0.562 |  |
|  | Group | 7.37E-04 | 3, 36 | 2.46E-04 | 0.250 | 0.860 | 0.020 |  |
| IPL-L | Time x Group | 8.25E-05 | 3, 36 | 2.75E-05 | 0.249 | 0.860 | 0.020 | 0.983 |
|  | Time | 3.35E-04 | 1, 36 | 3.35E-04 | 3.032 | 0.090 | 0.078 |  |
|  | Group | 3.60E-04 | 3, 36 | 1.20E-04 | 0.279 | 0.840 | 0.023 |  |
| SPL-R | Time x Group | 1.04E-03 | 3, 36 | 3.48E-04 | 4.207 | 0.010 | 0.260 | 0.04* |
|  | Time | 1.14E-03 | 1, 36 | 1.14E-03 | 13.720 | <0.001 | 0.276 |  |
|  | Group | 6.74E-04 | 3, 36 | 2.25E-04 | 0.246 | 0.860 | 0.020 |  |
| SPL-L | Time x Group | 7.00E-07 | 3, 36 | 2.33E-07 | 0.001 | >0.99 | 0.001 | >0.99 |
|  | Time | 3.82E-04 | 1, 36 | 3.82E-04 | 2.143 | 0.150 | 0.056 |  |
|  | Group | 2.53E-05 | 3, 36 | 8.42E-06 | 0.009 | >0.99 | 0.001 |  |
| SMA-R | Time x Group | 1.27E-04 | 3, 36 | 4.22E-05 | 1.543 | 0.220 | 0.114 | 0.587 |
|  | Time | 3.93E-04 | 1, 36 | 3.93E-04 | 14.370 | <0.001 | 0.285 |  |
|  | Group | 3.02E-05 | 3, 36 | 1.01E-05 | 0.028 | >0.99 | 0.002 |  |
| SMA-L | Time x Group | 4.23E-05 | 3, 36 | 1.41E-05 | 0.420 | 0.740 | 0.034 | >0.99 |
|  | Time | 9.93E-05 | 1, 36 | 9.93E-05 | 2.951 | 0.090 | 0.076 |  |
|  | Group | 8.60E-05 | 3, 36 | 2.87E-05 | 0.121 | 0.950 | 0.010 |  |
| M1-R | Time x Group | 4.98E-05 | 3, 36 | 1.66E-05 | 0.372 | 0.770 | 0.030 | >0.99 |
|  | Time | 2.22E-04 | 1, 36 | 2.22E-04 | 4.979 | 0.030 | 0.121 |  |
|  | Group | 6.27E-06 | 3, 36 | 2.09E-06 | 0.007 | >0.99 | 0.001 |  |
| M1-L | Time x Group | 1.29E-04 | 3, 36 | 4.30E-05 | 0.777 | 0.510 | 0.061 | >0.99 |
|  | Time | 1.76E-05 | 1, 36 | 1.76E-05 | 0.319 | 0.580 | 0.009 |  |
|  | Group | 9.96E-05 | 3, 36 | 3.32E-05 | 0.097 | 0.960 | 0.008 |  |

M1_L/R: left/right primary motor cortex, IPL_L/R: left/right inferior parietal lobule, SPL-L: left superior parietal lobule, SMA-R: right supplementary motor area. * *p* < 0.05, ** *p* < 0.01, *** *p* < 0.001

**Supplementary Table S6**

**RM-ANOVA results for the effects of intervention on FC of Network**

| **Network** | **ANOVA table** | **SS** | **DFn, DFd** | **MS** | ***F*** | ***P*** | **partial *η²*** | ***P*_adj (FDR)** |
| --- | --- | --- | --- | --- | --- | --- | --- | --- |
| IPL_R~SMA_R | Group x Time | 1.61E-01 | 3, 36 | 5.36E-02 | 6.343 | 0.001 | 0.346 | 0.028* |
|  | Time | 4.83E-02 | 1, 36 | 4.83E-02 | 5.718 | 0.020 | 0.137 |  |
|  | Group | 1.32E-01 | 3, 36 | 4.41E-02 | 0.834 | 0.480 | 0.065 |  |
| SMA_R~SMA_L | Group x Time | 7.93E-02 | 3, 36 | 2.64E-02 | 5.540 | 0.003 | 0.316 | 0.042* |
|  | Time | 3.89E-02 | 1, 36 | 3.89E-02 | 8.148 | 0.007 | 0.185 |  |
|  | Group | 7.79E-02 | 3, 36 | 2.60E-02 | 0.405 | 0.750 | 0.033 |  |
| IPL_R~IPL_L | Group x Time | 4.55E-02 | 3, 36 | 1.52E-02 | 0.213 | 0.890 | 0.017 | >0.99 |
|  | Time | 1.81E-01 | 1, 36 | 1.81E-01 | 2.541 | 0.120 | 0.066 |  |
|  | Group | 1.10E-02 | 3, 36 | 3.65E-03 | 0.034 | >0.99 | 0.003 |  |
| IPL_R~SPL_R | Group x Time | 1.56E-01 | 3, 36 | 5.19E-02 | 0.826 | 0.490 | 0.064 | >0.99 |
|  | Time | 1.08E-01 | 1, 36 | 1.08E-01 | 1.724 | 0.200 | 0.046 |  |
|  | Group | 3.18E-01 | 3, 36 | 1.06E-01 | 0.715 | 0.550 | 0.056 |  |
| IPL_R~SPL_L | Group x Time | 2.43E-01 | 3, 36 | 8.11E-02 | 1.491 | 0.230 | 0.110 | >0.99 |
|  | Time | 1.88E-02 | 1, 36 | 1.88E-02 | 0.346 | 0.560 | 0.010 |  |
|  | Group | 1.90E-01 | 3, 36 | 6.34E-02 | 0.658 | 0.580 | 0.052 |  |
| IPL_R~SMA_L | Group x Time | 3.87E-02 | 3, 36 | 1.29E-02 | 0.336 | 0.800 | 0.017 | >0.99 |
|  | Time | 1.83E-02 | 1, 36 | 1.83E-02 | 0.479 | 0.490 | 0.006 |  |
|  | Group | 1.03E-01 | 3, 36 | 3.44E-02 | 0.546 | 0.650 | 0.053 |  |
| IPL_R~M1_R | Group x Time | 5.51E-02 | 3, 36 | 1.84E-02 | 0.513 | 0.680 | 0.041 | >0.99 |
|  | Time | 5.91E-07 | 1, 36 | 5.91E-07 | 0.000 | >0.99 | 0.000 |  |
|  | Group | 6.02E-02 | 3, 36 | 2.01E-02 | 0.312 | 0.820 | 0.025 |  |
| IPL_R~M1_L | Group x Time | 3.80E-02 | 3, 36 | 1.27E-02 | 0.356 | 0.780 | 0.018 | >0.99 |
|  | Time | 6.84E-03 | 1, 36 | 6.84E-03 | 0.193 | 0.660 | 0.003 |  |
|  | Group | 3.45E-02 | 3, 36 | 1.15E-02 | 0.224 | 0.880 | 0.015 |  |
| IPL_L~SPL_R | Group x Time | 2.72E-02 | 3, 36 | 9.07E-03 | 0.143 | 0.930 | 0.012 | >0.99 |
|  | Time | 6.04E-02 | 1, 36 | 6.04E-02 | 0.955 | 0.330 | 0.026 |  |
|  | Group | 1.99E-02 | 3, 36 | 6.65E-03 | 0.057 | 0.980 | 0.005 |  |
| IPL_L~SPL_L | Group x Time | 1.49E-02 | 3, 36 | 4.98E-03 | 0.042 | 0.990 | 0.003 | >0.99 |
|  | Time | 1.21E-03 | 1, 36 | 1.21E-03 | 0.010 | 0.920 | 0.000 |  |
|  | Group | 5.60E-02 | 3, 36 | 1.87E-02 | 0.168 | 0.920 | 0.014 |  |
| IPL_L~SMA_R | Group x Time | 1.10E-02 | 3, 36 | 3.67E-03 | 0.077 | 0.970 | 0.006 | >0.99 |
|  | Time | 2.17E-03 | 1, 36 | 2.17E-03 | 0.045 | 0.830 | 0.001 |  |
|  | Group | 2.38E-02 | 3, 36 | 7.95E-03 | 0.105 | 0.960 | 0.009 |  |
| IPL_L~SMA_L | Group x Time | 1.65E-02 | 3, 36 | 5.51E-03 | 0.166 | 0.920 | 0.014 | >0.99 |
|  | Time | 3.37E-02 | 1, 36 | 3.37E-02 | 1.018 | 0.320 | 0.028 |  |
|  | Group | 5.94E-02 | 3, 36 | 1.98E-02 | 0.239 | 0.870 | 0.02 |  |
| IPL_L~M1_R | Group x Time | 3.90E-02 | 3, 36 | 1.30E-02 | 0.232 | 0.870 | 0.018 | >0.99 |
|  | Time | 1.67E-02 | 1, 36 | 1.67E-02 | 0.299 | 0.590 | 0.008 |  |
|  | Group | 2.26E-02 | 3, 36 | 7.52E-03 | 0.091 | 0.960 | 0.008 |  |
| IPL_L~M1_L | Group x Time | 1.03E-02 | 3, 36 | 3.45E-03 | 0.061 | 0.980 | 0.005 | >0.99 |
|  | Time | 1.06E-02 | 1, 36 | 1.06E-02 | 0.188 | 0.670 | 0.005 |  |
|  | Group | 5.49E-02 | 3, 36 | 1.83E-02 | 0.296 | 0.830 | 0.024 |  |
| SPL_R~SPL_L | Group x Time | 2.29E-01 | 3, 36 | 7.64E-02 | 1.138 | 0.350 | 0.087 | >0.99 |
|  | Time | 1.43E-03 | 1, 36 | 1.43E-03 | 0.021 | 0.880 | 0.001 |  |
|  | Group | 2.68E-01 | 3, 36 | 8.93E-02 | 0.811 | 0.500 | 0.063 |  |
| SPL_R~SMA_R | Group x Time | 1.46E-02 | 3, 36 | 4.88E-03 | 0.132 | 0.940 | 0.011 | >0.99 |
|  | Time | 1.10E-02 | 1, 36 | 1.10E-02 | 0.298 | 0.590 | 0.008 |  |
|  | Group | 2.88E-02 | 3, 36 | 9.59E-03 | 0.151 | 0.930 | 0.012 |  |
| SPL-R~SMA-L | Group x Time | 3.18E-01 | 3, 36 | 1.06E-01 | 3.674 | 0.020 | 0.234 | 0.187 |
|  | Time | 7.99E-02 | 1, 36 | 7.99E-02 | 2.768 | 0.100 | 0.071 |  |
|  | Group | 1.46E-01 | 3, 36 | 4.86E-02 | 0.602 | 0.620 | 0.048 |  |
| SPL_R~M1_R | Group x Time | 1.18E-01 | 3, 36 | 3.92E-02 | 0.698 | 0.560 | 0.055 | >0.99 |
|  | Time | 1.92E-02 | 1, 36 | 1.92E-02 | 0.341 | 0.560 | 0.009 |  |
|  | Group | 8.42E-02 | 3, 36 | 2.81E-02 | 0.379 | 0.770 | 0.031 |  |
| SPL_R~M1_L | Group x Time | 4.60E-02 | 3, 36 | 1.53E-02 | 0.322 | 0.810 | 0.026 | >0.99 |
|  | Time | 3.07E-03 | 1, 36 | 3.07E-03 | 0.064 | 0.800 | 0.002 |  |
|  | Group | 1.95E-02 | 3, 36 | 6.49E-03 | 0.081 | 0.970 | 0.007 |  |
| SPL_L~SMA_R | Group x Time | 6.03E-02 | 3, 36 | 2.01E-02 | 0.367 | 0.780 | 0.030 | >0.99 |
|  | Time | 3.71E-02 | 1, 36 | 3.71E-02 | 0.676 | 0.420 | 0.018 |  |
|  | Group | 7.83E-02 | 3, 36 | 2.61E-02 | 0.338 | 0.800 | 0.027 |  |
| SPL_L~SMA_L | Group x Time | 1.63E-01 | 3, 36 | 5.44E-02 | 1.467 | 0.240 | 0.109 | >0.99 |
|  | Time | 1.09E-02 | 1, 36 | 1.09E-02 | 0.293 | 0.590 | 0.008 |  |
|  | Group | 1.60E-01 | 3, 36 | 5.32E-02 | 0.686 | 0.570 | 0.054 |  |
| SPL_L~M1_R | Group x Time | 5.18E-02 | 3, 36 | 1.73E-02 | 0.421 | 0.740 | 0.034 | >0.99 |
|  | Time | 5.50E-03 | 1, 36 | 5.50E-03 | 0.134 | 0.720 | 0.004 |  |
|  | Group | 5.36E-02 | 3, 36 | 1.79E-02 | 0.186 | 0.910 | 0.015 |  |
| SPL_L~M1_L | Group x Time | 9.46E-02 | 3, 36 | 3.15E-02 | 0.530 | 0.660 | 0.042 | >0.99 |
|  | Time | 3.98E-02 | 1, 36 | 3.98E-02 | 0.668 | 0.420 | 0.018 |  |
|  | Group | 3.05E-02 | 3, 36 | 1.02E-02 | 0.110 | 0.950 | 0.009 |  |
| SMA-R~M1_R | Group x Time | 7.52E-02 | 3, 36 | 2.51E-02 | 3.714 | 0.020 | 0.236 | 0.140 |
|  | Time | 1.93E-01 | 1, 36 | 1.93E-01 | 28.500 | <0.001 | 0.442 |  |
|  | Group | 6.56E-03 | 3, 36 | 2.19E-03 | 0.033 | >0.99 | 0.003 |  |
| SMA_R~M1_L | Group x Time | 3.67E-02 | 3, 36 | 1.22E-02 | 0.286 | 0.830 | 0.023 | >0.99 |
|  | Time | 2.81E-02 | 1, 36 | 2.81E-02 | 0.660 | 0.420 | 0.018 |  |
|  | Group | 1.09E-02 | 3, 36 | 3.62E-03 | 0.058 | 0.980 | 0.005 |  |
| SMA_L~M1_R | Group x Time | 3.65E-02 | 3, 36 | 1.22E-02 | 0.434 | 0.730 | 0.035 | >0.99 |
|  | Time | 2.08E-02 | 1, 36 | 2.08E-02 | 0.741 | 0.390 | 0.020 |  |
|  | Group | 4.45E-02 | 3, 36 | 1.48E-02 | 0.235 | 0.870 | 0.019 |  |
| SMA_L~M1_L | Group x Time | 8.10E-02 | 3, 36 | 2.70E-02 | 0.746 | 0.530 | 0.059 | >0.99 |
|  | Time | 5.12E-03 | 1, 36 | 5.12E-03 | 0.142 | 0.710 | 0.004 |  |
|  | Group | 1.04E-01 | 3, 36 | 3.46E-02 | 0.547 | 0.650 | 0.44 |  |
| M1_R~M1_L | Group x Time | 5.49E-02 | 3, 36 | 1.83E-02 | 0.488 | 0.690 | 0.039 | >0.99 |
|  | Time | 1.67E-02 | 1, 36 | 1.67E-02 | 0.445 | 0.510 | 0.012 |  |
|  | Group | 5.40E-02 | 3, 36 | 1.80E-02 | 0.208 | 0.890 | 0.017 |  |

M1_L/R: left/right primary motor cortex, IPL_L/R: left/right inferior parietal lobule, SPL-L/R: left/right superior parietal lobule, SMA-L/R: left/right supplementary motor area. * *p* < 0.05, ** *p* < 0.01, *** *p* < 0.001

**Supplementary Table S7.**

**Change-score 2 × 2 factorial ANOVA testing nGVS × tDCS interaction effects**

| **Variables** | **Δ Sham** | **Δ nGVS** | **Δ tDCS** | **Δ nGVS+tDCS** | **nGVS effect *F*(*p*)** | **tDCS effect *F*(*p*)** | **nGVS × tDCS interaction *F*(*p*)** | **Interaction estimate (95% CI)** | **Interpretation** |
| --- | --- | --- | --- | --- | --- | --- | --- | --- | --- |
| Path length (cm) | 21.319 ± 173.837 | -169.392 ± 153.969 | -143.288 ± 86.554 | -349.421 ± 122.839 | *F*(1,36) = 20.585,  *p* = <0.001 | *F*(1,36) = 15.525,  *p* = <0.001 | *F*(1,36) = 0.031,  *p* = 0.861 | -15.423  [-192.816, 161.971] | NS |
| Area (cm²) | -2.475 ± 636.798 | -1165.170 ± 1056.968 | -871.922 ± 588.789 | -1205.745 ± 954.940 | *F*(1,36) = 8.052,  *p* = 0.007 | *F*(1,36) = 2.978,  *p* = 0.093 | *F*(1,36) = 2.470,  *p* = 0.125 | 828.872  [-240.700, 1898.445] | NS |
| GM_L | 0.020±0.045 | -0.023±0.022 | -0.015±0.019 | -0.031±0.032 | *F*(1,36) = 8.967,  *p* = 0.005 | *F*(1,36) = 4.963,  *p* = 0.032 | *F*(1,36) = 1.867,  *p* = 0.180 | 0.0272 [-0.013, 0.068] | NS |
| GM_R | 0.005±0.033 | -0.026±0.017 | -0.013±0.018 | -0.032±0.024 | *F*(1,36) = 10.964,  *p* = 0.002 | *F*(1,36) = 2.498,  *p* = 0.123 | *F*(1,36) = 0.599,  *p* = 0.444 | 0.012 [-0.019, 0.042] | NS |
| IPL_R (mmol/L) | 0.006±0.010 | 0.005±0.013 | 0.018±0.013 | 0.022±0.011 | *F*(1,36) = 0.200,  *p* = 0.657 | *F*(1,36) = 15.311,  *p* = <0.001 | *F*(1,36) = 0.298,  *p* = 0.589 | 0.004 [-0.011, 0.019] | NS |
| SPL_R (mmol/L) | -0.004±0.006 | 0.008±0.017 | 0.009±0.014 | 0.016±0.010 | *F*(1,36) = 5.616,  *p* = 0.023 | *F*(1,36) = 6.648,  *p* = 0.014 | *F*(1,36) = 0.358,  *p* = 0.553 | -0.005 [-0.021, 0.012] | NS |
| IPL_R and SMA_R | -0.032±0.105 | -0.048±0.190 | 0.143±0.064 | 0.134±0.128 | *F*(1,36) = 0.099,  *p* = 0.755 | *F*(1,36) = 18.923,  *p* = <0.001 | *F*(1,36) = 0.006,  *p* = 0.940 | 0.006 [-0.160, 0.173] | NS |
| SMA-R and SMA_L | -0.055±0.076 | 0.053±0.095 | 0.059±0.105 | 0.120±0.111 | *F*(1,36) = 7.470,  *p* = 0.010 | *F*(1,36) = 8.552,  *p* = 0.006 | *F*(1,36) = 0.600,  *p* = 0.444 | -0.048 [-0.173, 0.077] | NS |

Values are presented as mean ± SD. Change scores (Δ) were calculated as Post − Pre. The four arms were recoded into a 2 × 2 factorial structure with nGVS (No/Yes) and tDCS (No/Yes) as between-subject factors. Interaction estimate = ΔnGVS+tDCS − ΔnGVS − ΔtDCS + Δsham; 95% CI = 95% confidence interval for this estimate. Negative Δ values indicate post-intervention reductions for COP and EMG RMS outcomes. FC values are Fisher-z-transformed correlation coefficients. Interpretation indicates whether the nGVS × tDCS interaction was statistically significant. NS = non-significant interaction, i.e., no statistical evidence for a synergistic effect between nGVS and tDCS.

**Supplementary Table S8.**

Summary of excluded fNIRS channels after channel-quality inspection

| **Group** | **Time** | **Excluded channels, n (%)** |
| --- | --- | --- |
| Sham | Pre | 11 / 220 (5.0%) |
| nGVS | Pre | 8 / 220 (3.6%) |
| HD-tDCS | Pre | 9 / 220 (4.1%) |
| nGVS + HD-tDCS | Pre | 6 / 220 (2.7%) |
| Sham | Post | 10 / 220 (4.5%) |
| nGVS | Post | 10 / 220 (4.5%) |
| HD-tDCS | Post | 12 / 220 (5.5%) |
| nGVS + HD-tDCS | Post | 11 / 220 (5.0%) |

Each group included 10 participants, and 22 fNIRS channels were assessed per participant at each time point, yielding 220 channels per group per time point.
